# Unlocking the Chemical and Structural Complexity of Aluminum Hydroxy Acetates: from Commodity Chemicals to Porous Materials

**DOI:** 10.1002/chem.202403634

**Published:** 2024-11-22

**Authors:** Bastian Achenbach, Lena‐Marie Liedtke, Christian Näther, Erik Svensson Grape, A. Ken Inge, Norbert Stock

**Affiliations:** ^1^ Institute of Inorganic Chemistry Kiel University Max-Eyth-Str. 2 24118 Kiel Germany; ^2^ Department of Materials and Environmental Chemistry Stockholm University Stockholm Sweden; ^3^ Current address: Department of Chemistry and Biochemistry Material Science Institute University of Oregon Eugene Oregon 97403 United States; ^4^ Wallenberg Initiative Materials Science for Sustainability Department of Materials and Environmental Chemistry Stockholm University Stockholm Sweden; ^5^ Kiel Nano, Surface and Interface Science KiNSIS Kiel University Christian-Albrechts-Platz 4 24118 Kiel Germany

**Keywords:** High-throughput screening, Adsorption, Aluminum carboxylates, Porosity, Green synthesis

## Abstract

Aluminum acetates have been in use for more than a century, but despite their widespread commercial applications, essential scientific knowledge of their synthesis‐structure‐property relationships is lacking. High‐throughput screening, followed by fine tuning and extensive optimization of reaction conditions using Al^3+^, OH^−^ and CH_3_COO^−^ ions, has unraveled their complex synthetic chemistry, yielding for the first time the four phase pure products Al(OH)(O_2_CCH_3_)_2_ ⋅ x H_2_O (x=0, 2) (**1A** and CAU‐65, **1B**), Al_3_O(HO_2_CCH_3_)(O_2_CCH_3_)_7_ (**2**), and the porous aluminum salt [Al_24_(OH)_56_(CH_3_COO)_12_](OH)_4_ (CAU‐55‐OH, **3**). Structure determination by electron and X‐ray diffraction was carried out and the data suggested porosity for **1B** and **3**, which was confirmed by physisorption experiments. Even the scale‐up to the 10 L scale was accomplished for **1A**, **1B** and **3** with yields of up to 1.1 kg (99 %). This study of a seemingly simple chemical system provides important information on both fundamental inorganic chemistry and porous materials.

## Graphical abstract

A high‐throughput investigation involving more than 330 reactions in the system Al^3+^/CH_3_COOH/NaOH/H_2_O permitted the elucidation of the synthesis‐structure relationship and allowed us to shed light on the structural chemistry and porosity of aluminum acetates for the first time. The robust synthesis procedures permitted scale‐up under mild reaction conditions in high yields.

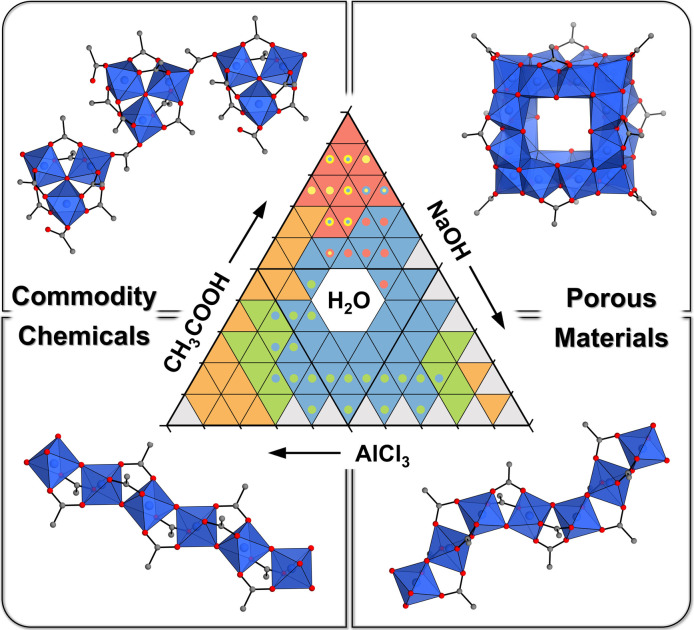

## Introduction

Despite the immense importance of aluminum and its widespread uses,^[1]^ the structures of many of its commercially used compounds remain unknown. Many synthetic aluminum compounds, produced on a large scale, including aluminum oxides/hydroxides,[^2^, ^3^, ^4^] zeolites[^5^, ^6^] and layered double hydroxides[^7^, ^8^, ^9^] which are utilized in a variety of applications. For instance, they are employed as abrasives,[^10^, ^11^, ^12^] catalyst supports[^9^, ^13^, ^14^] and mordants in dyeing.[^15^, ^16^]

Aluminum carboxylates as a class of aluminum compounds have gained increasing interest in the last decades, especially with the use of polycarboxylic acids for the synthesis of porous metal‐organic frameworks (MOFs).[^17^, ^18^] The combination of high chemical and thermal stability, readily available and inexpensive starting materials, and permanent porosity makes Al‐MOFs interesting candidates for a wide range of applications, including gas storage and separation,[^19^, ^20^] as well as heat exchange processes.[^21^, ^22^, ^23^]

While many polycarboxylic acids have been employed for the synthesis of Al‐MOFs,^[17]^ only relatively few in‐depth studies have been reported with monocarboxylic acids. In recent years aluminum monocarboxylates, denoted as AlOCs−X (aluminum oxo clusters, X=1–199) have been described mainly with arene monocarboxylic acid derivatives. Solvothermal reactions in the presence of organic solvents were used and resulted in the formation of Al−O clusters and Al−O nanorings.[^24^, ^25^]

Short‐chain aliphatic monocarboxylic acids have also been employed in the synthesis of aluminum carboxylates, which have been utilized industrially for over 140 years.[^26^, ^27^, ^28^, ^29^, ^30^, ^31^, ^32^] While basic aluminum formates Al(OH)_3‐x_(O_2_CH)_x_ (X=1, 2) have been used historically in the manufacture of water‐repellent textiles,^[33]^ aluminum triformate has recently been introduced as a promising material for carbon capture applications.^[20]^ Aluminum acetates are also commercially important and are used as metal sources for aluminum compounds or, for instance, in the pharmaceutical industry due to their antiperspirant, antimicrobial, and astringent properties, or as flame retardants.[^34^, ^35^] Despite being produced and used on an industrial scale, limited information on the syntheses, composition, and crystal structures of phase pure aluminum acetates is available.

Here we present the results of a systematic high‐throughput investigation of the system Al^3+^/NaOH/CH_3_COOH/H_2_O, which for the first time yielded four phase‐pure compounds: two pseudopolymorphs of composition Al(OH)(O_2_CCH_3_)_2_ ⋅ x H_2_O (x=0, 2) (**1A** and **1B/CAU‐65**), Al_3_O(HO_2_CCH_3_)(O_2_CCH_3_)_7_ (**2**) and the aluminum salt [Al_24_(OH)_56_(CH_3_COO)_12_](OH)_4_ (CAU‐55‐OH, **3**), unravelling the chemical and structural complexity of aluminum acetates and adding synthetic and structural information to fundamental inorganic chemistry and the field of porous materials.

## Results and Discussion

### Synthesis

**Figure 1:**
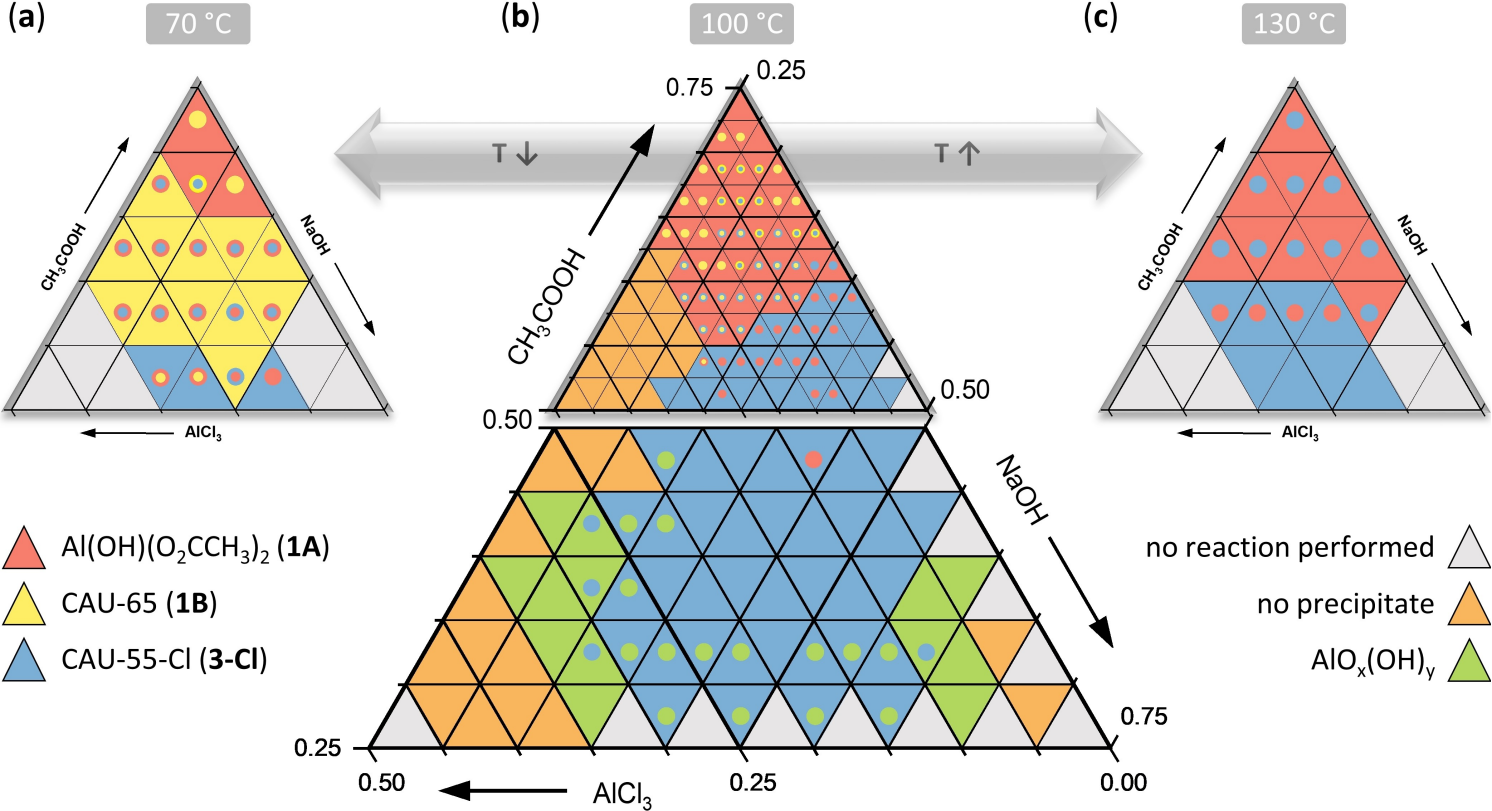
Ternary crystallization diagrams of crystalline phases observed in the chemical system AlCl_3_/CH_3_COOH/NaOH/H_2_O for reactions carried out at (**a**) 70 °C, (**b**) 100 °C and (**c**) 130 °C using high‐throughput methods. The observed phases are color‐coded, with the dominating phases represented by triangles and phase mixtures represented by colored circles. The grey triangles mark the parameter spaces that were not investigated.

Building on previously reported information,^[36]^ a new high‐throughput investigation of the chemical system Al^3+^/CH_3_COOH/NaOH/H_2_O was carried out followed by fine‐tuning and optimization of reaction conditions involving more than 330 individual reactions (Section S2).^[37]^ This allowed us to establish robust synthesis conditions leading to four metal acetates. The reaction temperature and the molar ratios of the reactants were systematically varied between 70 and 130 °C and 0.5–4 (AlCl_3_), 3–14 (CH_3_COOH) and 3–9 (NaOH) equivalents (Table S2.1–S2.9), respectively. The results are presented in Figure 1.

The lower part of Figure 1 visualizes the results obtained, when lower amounts of CH_3_COOH are used. At high NaOH or Al^3+^ concentrations, different polymorphs of AlO_x_(OH)_y_ (Boehmite, Gibbsite and Bayerite)^[38]^ (Figure S2.1) or clear solutions with no precipitate are observed (Figure 1b, green or dark grey triangles, respectively). Under a wide range of equimolar ratios of NaOH and CH_3_COOH, CAU‐55‐Cl (**3‐Cl**), a porous salt of composition [Al_24_(OH)_56_(O_2_CCH_3_)_12_]Cl_4_ was found (Figure 1b, lower part, blue triangles).^[36]^

The upper part of Figure 1 contains the results obtained at high acetic acid concentrations. The three diagrams summarize the results of identical reactions carried out at the three different reaction temperatures 70, 100 and 130 °C (Figure 1a, 1b, and 1c). At 70 and 100 °C (Figure 1a and b) the two pseudopolymorphs Al(OH)(O_2_CCH_3_)_2_ **1A** (red triangles) and Al(OH)(O_2_CCH_3_)_2_ ⋅ x H_2_O **1B/CAU‐65** (x=0–2) (yellow triangles) are found. They differ in the connectivity of the [AlO_6_] polyhedra and **1A** is the dominating pseudopolymorph over a wide range of molar ratios. The second pseudopolymorph Al(OH)(O_2_CCH_3_)_2_ ⋅ 2 H_2_O (CAU‐65, **1B**) is preferentially formed at higher Al^3+^ concentrations and low reaction temperatures. In contrast to **1A**, **1B** could not be obtained phase‐pure during our initial HT screening but this was accomplished in a subsequent HT investigation. These studies were carried out in a temperature gradient HT oven, employing different aluminum salts (NaAlO_2_, Al_2_(SO_4_)_3_ and Al(NO_3_)_3_) at a fixed composition and varying the reaction temperature and time (25 °C< T <100 °C, T=24, 48, 72 and 96 h). Thus, phase pure products of **1B** could be obtained at high concentrations of Al_2_(SO_4_)_3_ and NaOH (optimized molar ratios Al^3+^ : CH_3_COOH : NaOH=3 : 12 : 10) at a reaction temperature below 60 °C (t=20 h) (Figure S2.3, Table S2.10). Surprisingly, the use of NaAlO_2_ as the Al^3+^ source resulted in the formation of yet another aluminum hydroxide acetate with a composition of [Al_24_(OH)_56_(O_2_CCH_3_)_12_](OH)_4_ (**3**) which contains cationic Al_24_‐hydroxide‐acetate clusters (Table S2.15).^[36]^

**Figure 2:**
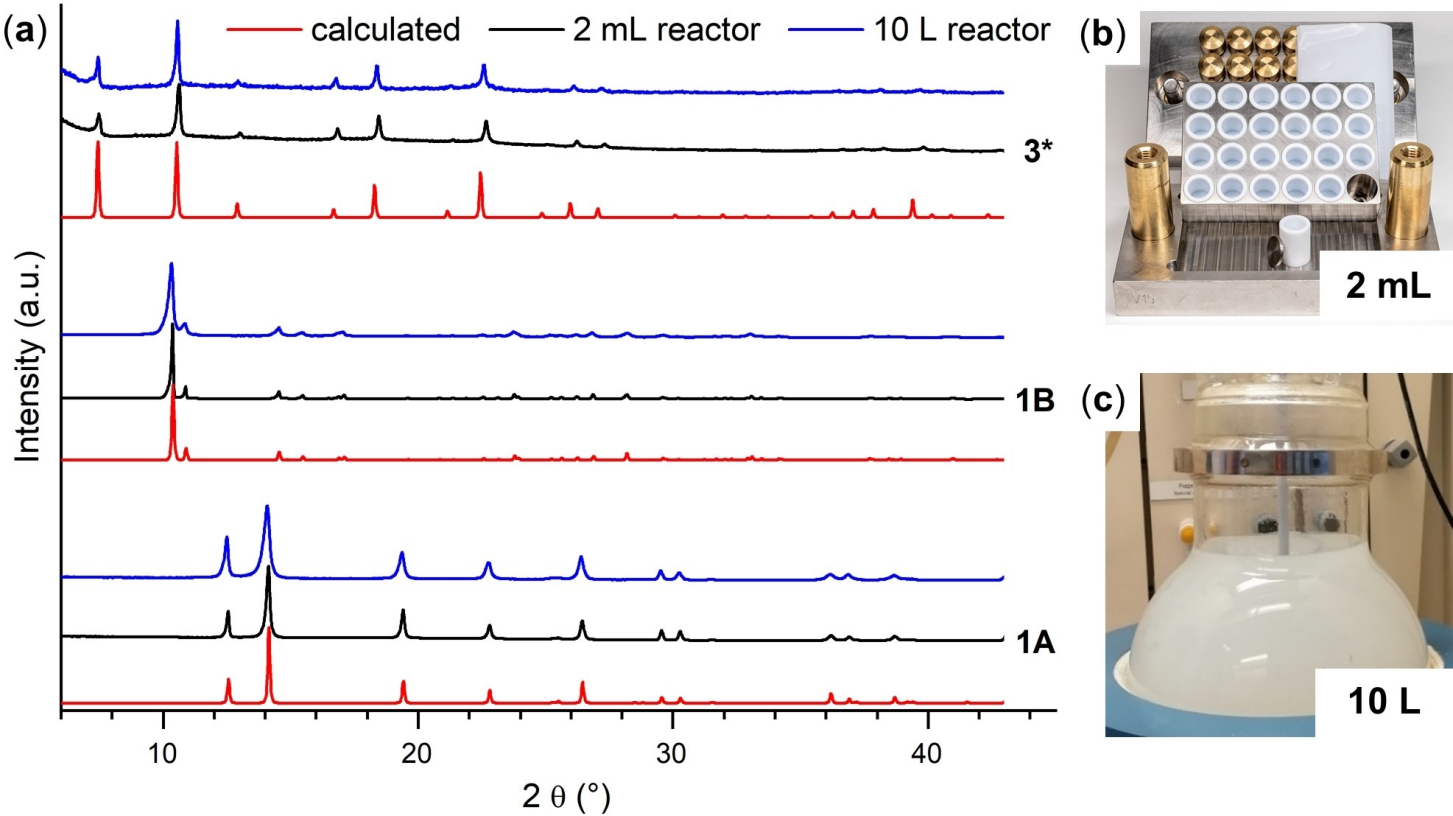
(**a**) Measured and calculated (red line) PXRD patterns of Al(OH)(O_2_CCH_3_)_2_ (**1A**) (bottom), CAU‐65 (**1B**) (middle) and CAU‐55‐OH (**3**) (top) synthesized in 2 mL high‐throughput (HT) reactors (black line) and in a 10 L reactor (blue line). (*) For **3**, the calculated PXRD pattern of CAU‐55‐Cl^[36]^ is shown. (**b**, **c**) Reactor types and set‐ups used in the discovery and synthesis scale up investigation. (**b**) 24 multiclave with 2 mL Teflon inserts, (**c**) 10 L round‐bottom flask with stirrer.

The in‐depth knowledge of the role of synthetic parameters on the product formation allowed us to establish scale‐up procedures for the aluminum hydroxy acetates (**1A**, **1B** and **3**) by step‐wise increasing the size of the batch reactor from 1 mL to 10 L (Section S2.2). A comparison of measured and calculated powder X‐ray diffraction (PXRD) patterns of the three aluminum hydroxy acetates synthesized at 1 mL and 10 L scale and the reactor employed in the scale‐up is shown in Figure 2. Reactions in the 10 L reactor yielded 0.3 kg/ 99 % and 1.1 kg/ 96 % of **1A** and **1B**, respectively. In contrast, maximum yields of less than 15 % were obtained for CAU‐55‐OH (**3**).^[39]^

The aluminum triacetate Al(O_2_CCH_3_)_3_ is another possible phase in the system Al^3+^/CH_3_COO^−^/OH^−^. It has already been postulated in 1938 but no structure has yet been reported.[^40^, ^41^, ^42^] Hence a study using synthetic procedures reported in 1950^[41]^ and 1956^[42]^ employing aluminum isopropoxide, glacial acetic acid and acetic anhydride as the solvent was carried out (Section 2.1.3). The results summarized in Figure S2.6 show that the molar ratio of glacial acetic acid to acetic anhydride has a strong impact on the product formation. At low concentrations of acetic anhydride compound **1A** is obtained, while at high concentrations a phase of composition Al_3_O(HO_2_CCH_3_)(O_2_CCH_3_)_7_ (**2**) could be isolated in the form of single crystals suitable for structure determination by X‐ray diffraction (Section S3, Figure S3.7). Other reported synthesis procedures resulted in the same microcrystalline phase as demonstrated by powder X‐ray diffraction. Based on reported PXRD data from literature, our investigation shows that the postulated aluminum triacetate Al(O_2_CCH_3_)_3_ is in fact Al_3_O(HO_2_CCH_3_)(O_2_CCH_3_)_7_ (**2**).^[41]^

### Crystal Structure

**Figure 3:**
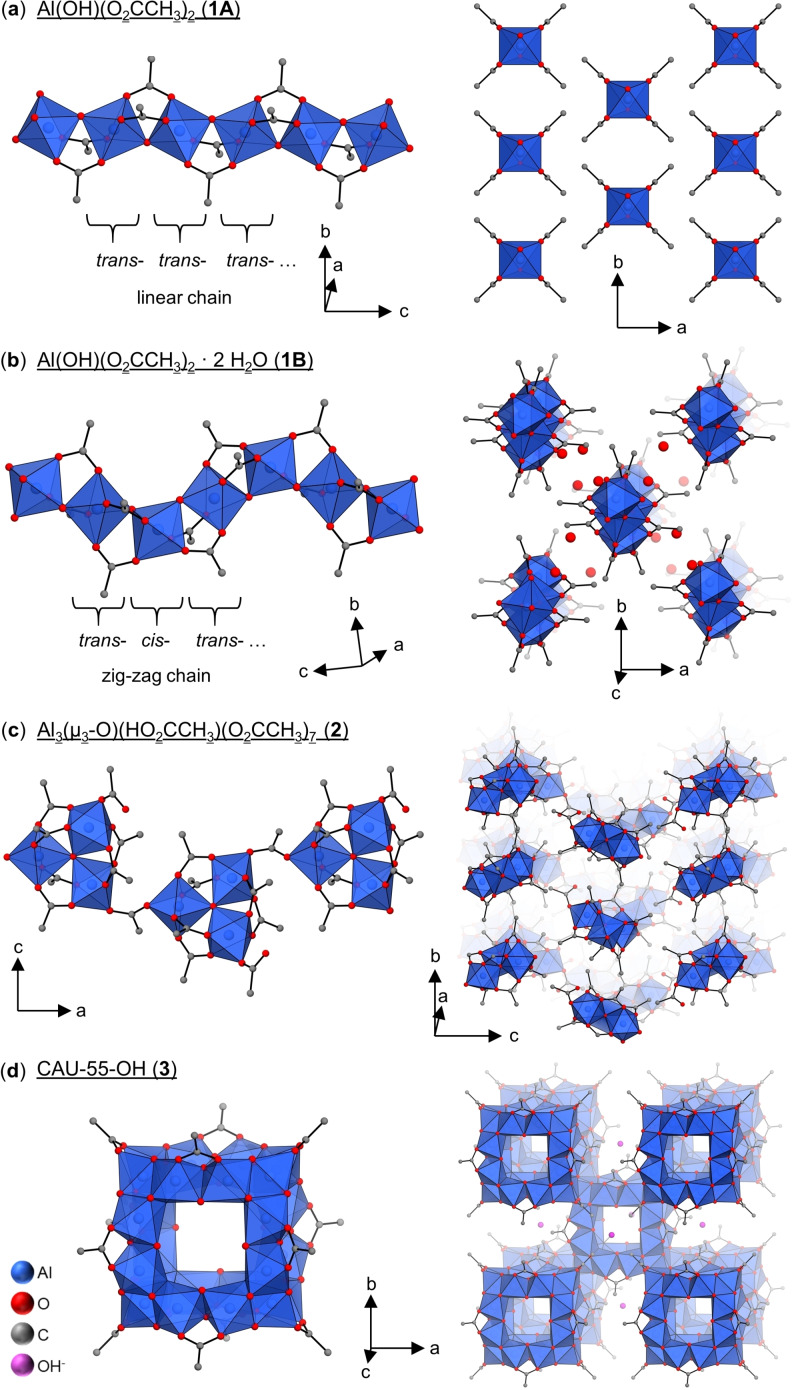
(**a**) Crystal structure of Al(OH)(O_2_CCH_3_)_2_ (**1A**). (**left**) 1D linear rod‐shaped building unit formed by *trans*‐corner‐sharing [AlO_6_]‐octahedra connected through *μ*‐OH‐groups. (**right**) Crystal structure of **1A** as seen along the *c*‐axis. (**b**) Crystal structure of CAU‐65 (**1B**). (**left**) 1D zigzag‐chains formed by alternating *cis*‐ and *trans*‐corner‐sharing [AlO_6_]‐octahedra connected through *μ*‐OH‐groups. (**right**) Crystal structure of **1B** as seen along the *c*‐axis. H_2_O molecules are shown which can be reversibly removed. (**c**) Crystal structure of Al_3_(O)(HO_2_CCH_3_)(O_2_CCH_3_)_7_ (**2**). (**left**) Chain of trinuclear *μ*_3_‐O‐centered building units composed of three corner‐sharing [AlO_6_]‐octahedra connected through six acetate groups. (**right**) Crystal structure of **2** as seen along the *a*‐axis. (**d**) Crystal structure of CAU‐55‐OH (**3**). (**left**) Al_24_ cluster ion {[Al_24_(OH)_56_(O_2_CCH_3_)_12_]^4+^} composed of eight edge‐sharing trinuclear building units occupying the corners of a cube. (**right**) Body centered packing of Al_24_ cluster cations and OH^−^ ions occupying the octahedral voids.

In the following paragraphs the main structural aspects of the four aluminum acetates are summarized. Further details are given in the supporting information (Section S3). The pseudopolymorphs of Al(OH)(O_2_CCH_3_)_2_ ⋅ x H_2_O (x=0, 2) (**1A** and **1B**) were obtained as microcrystalline products and structure determination was carried out by three‐dimensional electron diffraction (3D ED) followed by Rietveld refinement against PXRD data (Section S3.1–S3.3). The structures of **1A** and **1B** differ mainly in the connectivity of the [AlO_6_] octahedra, which lead to rod‐shaped building units. Compound **1A** contains the well‐known chains of *trans*‐ *μ*‐OH linked [AlO_6_] octahedra, which are also observed in the Al‐MOFs Al‐MIL‐53,[^43^, ^44^] Al‐CAU‐11^[45]^ and Al‐MIL‐68,^[46]^ where these are connected through dicarboxylate ions to form three‐dimensional networks. The formal replacement of dicarboxylate by acetate ions results in dense packing of the chains in **1A** (Figure 3b). The crystal structure of the second polymorph, Al(OH)(O_2_CCH_3_)_2_ ⋅ 2 H_2_O (CAU‐65, **1B**) is also composed of infinite chains of *μ*‐OH‐linked [AlO_6_] octahedra. However, zigzag chains are formed by alternating *cis*‐ and *trans*‐linked octahedra (Figure 3c), similar to the ones reported for MOF−303.^[47]^ The slight structural difference in the inorganic building unit has a strong impact on the properties. In the crystal structure of **1B**, the stacking of the chains of *cis*‐*trans*‐linked AlO_6_ polyhedra leads to the formation of one‐dimensional channels, which are occupied by two water molecules per formula unit (Figure 3f).

The crystal structure of the new aluminum acetate Al_3_O(HO_2_CCH_3_)(O_2_CCH_3_)_7_ (**2**) was determined by single‐crystal X‐ray diffraction (SCXRD) and phase purity of the bulk material was confirmed by a Rietveld refinement (Section S3.1 and S3.4). The structure of the coordination polymer **2** contains the well‐known trinuclear *μ*_3_‐O centered building units (M_3_(*μ*_3_−O)) consisting of three corner‐sharing [MO_6_] octahedra (M=Fe, Cr, Al, Sc, …) that are coordinated by six acetate groups, leading to the composition (M_3_(*μ*_3_−O)(O_2_CCH_3_)_6_)^+^.[^48^, ^49^, ^50^] In **2** each trimeric building unit contains one end‐on‐binding acetic acid molecule, which forms hydrogen bonds with neighboring oxygen atoms of the acetate groups. These trinuclear units are bridged by acetate ions resulting in zig−zag chains (Figure 3c).

CAU‐55‐OH (**3**) was obtained as nanocrystalline powder (d≤100 nm; Figure S3.9a). Based on the PXRD data (Figure S3.8) and composition **3** is isostructural to CAU‐55‐X, a series of porous compounds previously reported by our group with X=Cl, Br, I, HSO_4_.^[36]^ The crystal structure contains cationic Al_24_‐hydroxide‐acetate clusters of composition [Al_24_(OH)_56_(O_2_CCH_3_)_12_]^4+^ (Figure 3d), which are arranged in a body‐centered cubic packing. The voids between the Al_24_ clusters are occupied by counter ions and water molecules, which can be removed reversibly. For CAU‐55‐OH (**3**), a Le Bail fit was carried out to confirm the phase purity (Figure S3.4, Table S3.2), showing similar lattice parameters to those of previously described CAU‐55‐X materials, but only slightly contracted due to the incorporation of the relatively small hydroxide counter ions (Figure 2). Due to the data quality a Rietveld refinement could not be carried out successfully.

### Characterization

All compounds have been thoroughly characterized to confirm phase purity as well as to investigate their thermal and sorption properties.

IR spectroscopy confirms the presence of carboxylate groups and hydroxide groups due to their characteristic vibrational bands (Section S4.1). For Al_3_O(HO_2_CCH_3_)(O_2_CCH_3_)_7_ (**2**), additional vibrational bands at 1713 cm^−1^ are present in the IR spectrum, confirming the presence of coordinating acetic acid molecules (Section S4.1). The high water content of the CAU‐55‐OH (**3**) sample correlates with a high intensity of the broad band in the region of 3000–3700 cm^−1^. For Al(OH)(O_2_CCH_3_)_2_ (**1A**) a sharp vibrational band is observed in at 3700 cm^−1^, which is typical for bridging *μ*‐OH groups.^[51]^ However, the specific band of the bridging OH groups in **1B** is overlapped by several broad bands in the region of 3000–3700 cm^−1^ due to the formation of hydrogen bonds between the *μ*‐OH groups and the water molecules in the pores.

Elemental analysis in combination with thermogravimetric measurements allowed the determination of the water content and the overall composition of the samples (Section S4.2 and S4.3). The results of the elemental analyses are in good agreement with the calculated carbon and hydrogen contents of the compounds. For CAU‐55‐OH (**3**), the absence of any other anion than OH^−^ possibly present as an impurity was further confirmed by EDX analyses (Table S4.2).

**Figure 4:**
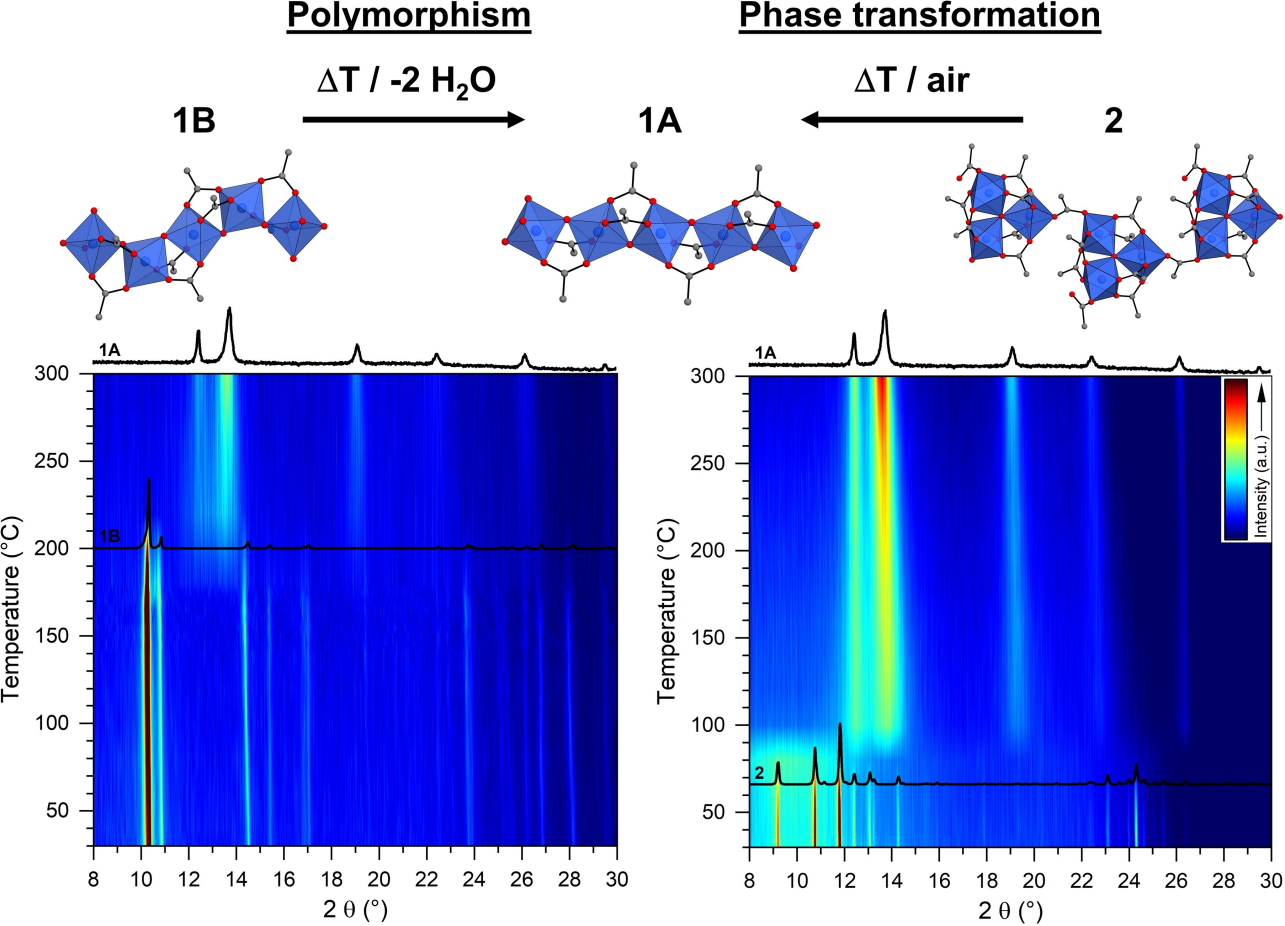
Contour plots of the VT‐PXRD data and PXRD data of different crystalline phases observed during VT‐PXRD measurements of CAU‐65 (**1B**) (**left**) and Al_3_(O)(HO_2_CCH_3_)(O_2_CCH_3_)_7_ (**2**) (**right**).

Thermogravimetric measurements (Figure S4.2–S4.5) in combination with variable‐temperature powder X‐ray diffraction (VT‐PXRD) were used to determine the solvent content, decomposition temperatures and temperature‐dependent phase transformations. The first steps (room temperature to approximately 150 °C) can be attributed to the removal of water molecules from the pores of CAU‐65 (**1B**) and CAU‐55‐OH (**3**) resulting in small changes in the reflection positions and relative intensities in the powder patterns (Figure 4, Figure S4.6–S4.7). For CAU‐65 (**1B**) and Al_3_O(HO_2_CCH_3_)(O_2_CCH_3_)_7_ (**2**), a phase transformation to Al(OH)(O_2_CCH_3_)_2_ (**1A**) by rearrangement of the inorganic building unit is observed in the range of 180–220 °C and 70–120 °C respectively in the VT‐PXRD measurements using open capillaries (Figure 4 and S4.6–S4.8). Thus, **1A** can be assigned to be the thermodynamically stable product under these conditions. Subsequent decomposition of **1A** occurs at temperatures above 250 °C (Figure S4.2). CAU‐55‐OH (**3**) shows a lower thermal stability compared to the other CAU‐55 derivatives (180–200 °C) with a decomposition temperature of ~110 °C (Figure S4.5).^[36]^ Further heating to 800 °C leads to the decomposition of the compounds and the formation of a reaction product of low crystallinity, which can be assigned to Al_2_O_3_.

**Figure 5:**
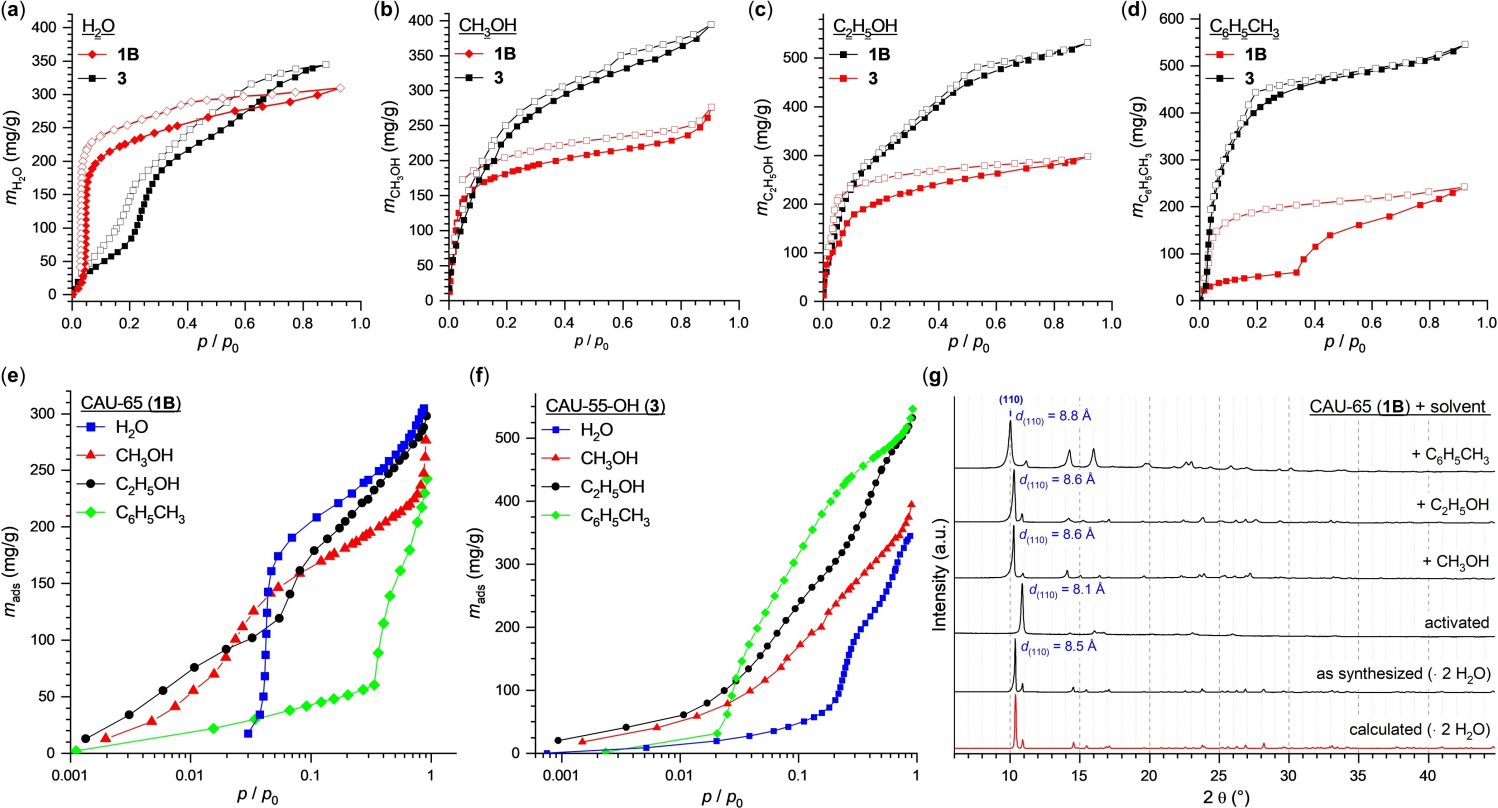
(**a**) – (**d**) Water (H_2_O) (**a**), methanol (CH_3_OH) (**b**), ethanol (C_2_H_5_OH) (**c**) and toluene (C_6_H_5_CH_3_) (**d**) sorption isotherms of CAU‐65 (**1B**) and CAU‐55‐OH (**3**) collected at 298 K. Prior to the measurements the samples were activated at 70 °C under reduced pressure. (**e**) and (**f**) Vapor sorption isotherms of CAU‐65 (**1B**) (**e**) and CAU‐55‐OH (**3**) (**f**) collected at 298 K and plotted with a logarithmic x‐axis. Prior to the sorption measurements the samples were activated at 70 °C under reduced pressure. (**g**) Powder X‐ray diffraction data of **1B** after the adsorption of different organic vapors leading to an expansion of the lattice parameters, i. e., a larger unit cell indicated by a shift of the 110 Bragg reflection (shown in blue), corresponding to a change in distances between the Al−O chains. Prior to the PXRD measurements, the samples were activated at 70 °C under reduced pressure then soaked in methanol (CH_3_OH), ethanol (C_2_H_5_OH) or toluene (C_6_H_5_CH_3_). Additionally, the measured and calculated PXRD patterns of the as‐synthesized product of **1B** containing water as the guest molecules are shown.

Based on the crystal structures and the previously described analytical data, porosity of **1B** and **3** can be anticipated due to the water content and the structural integrity upon dehydration. Hence, N_2_ and H_2_O isotherms were recorded at 77 and 298 K, respectively (Figure 5, Figure S5.1–S5.3). Prior to the sorption measurements, the samples were activated under reduced pressure at elevated temperature (70 °C). The stability of the samples after the sorption measurements was confirmed by PXRD (Figure S5.5–S5.8). As anticipated, the N_2_ sorption isotherms of **1B** and **3** show a Type I shape up to *p*/*p*_0_ ≈0.1, which is typical for microporous materials, whereas **1A** and **2** show no porosity against nitrogen (Figure S5.1). The slope at higher *p*/*p*_0_ values and the small hysteresis of the N_2_ sorption isotherm of CAU‐55‐OH can be attributed to textural porosity due to nano‐sized particles (Figure S3.9). The hysteresis in the sorption isotherm of **1B** can be rationalized by the small pore diameter and kinetic effects due to the low measurement temperature. The evaluation of the N_2_ sorption data results in BET surface areas of *S*_BET_=146 and 1005 m^2^/g for **1B** and **3**, respectively.

The water sorption for CAU‐65 (**1B**) leads to an S‐shaped isotherm with a sharp increase at *p*/*p*_0_ ≈0.04 and a water uptake of 200 mg/g at *p*/*p*_0_=0.1 (Figure 5a) corresponding to two water molecules per formula unit, which is in line with the results obtained from crystal structure determination (Figure 3). Such S‐shaped water isotherms have been reported for Al‐MOFs and are structurally related to the formation of hydrogen bonds between water molecules and the framework.[^52^, ^53^] In addition to water sorption measurement, the adsorption of organic vapors was investigated for **1B** and **3** (Figure 5, Figure S5.4). For methanol, ethanol and toluene an adsorption capacity between 250 and 300 mg/g corresponding to ~1 mol/mol per formula unit was observed for **1B** (Figure 5b – d). With increasing hydrophobicity of the probe molecule, a shift of the adsorption curve to higher *p*/*p*_0_ and an increased hysteresis between adsorption and desorption was observed, demonstrating the hydrophilic behavior of **1B**. The removal of water and subsequent adsorption of organic vapors also results in an expansion of the lattice parameters, i. e., a larger unit cell indicated by a shift of the 110 Bragg reflection (Δ*d*_(110)_≤0.7 Å), and variations in the relative intensities of the reflections of the PXRD pattern of **1B** (Table S5.1, Figure 5e, Figure S5.5–S.8).

The water sorption isotherm of CAU‐55‐OH (**3**) (Figure 5a) exhibits an S‐shaped isotherm similar to the other CAU‐55‐X samples with a maximum uptake capacity of 385 mg/g. In contrast to **1B**, a shift of the adsorption curve to lower *p*/*p*_0_ was observed for **3** with increasing hydrophobicity of the probe molecule, demonstrating the more hydrophobic behavior of **3**. The slope at higher *p*/*p*_0_ and the small hysteresis of the vapor sorption isotherm of CAU‐55‐OH (**3**) can be attributed to textural porosity due to nano‐sized particles (Figure S3.9).

## Conclusions

Since their first documented use more than 140 years ago, the synthetic and structural chemistry of aluminum acetates has been unraveled. Utilizing cheap, readily available, and environmentally benign commodity chemicals, the HT study has identified the fields of formation of four phase‐pure products, two of which are permanently porous. Subsequently, scalable green synthesis procedures using short reaction times and affording high yields (up to 1.1 kg/96 % for the porous compound CAU‐65) were developed. The crystal structures of the four aluminum acetates were elucidated through 3D electron diffraction or single‐crystal X‐ray diffraction measurements, followed by Rietveld‐refinement against PXRD data. Fine tuning of the synthesis conditions allowed us to direct the formation of chains of AlO_6_ polyhedra with either *trans* or *cis‐trans* connectivity (**1A** and **1B**), trinuclear Al−O clusters, or cationic Al_24_ clusters, which are the decisive factor for the porosity of the compounds. Based on the crystal structures, porosity of **1B** and **3** was anticipated and also confirmed by N_2_ and vapor sorption experiments. Using vapors of different hydrophilicity, it was shown that **3** exhibits a higher affinity for hydrophobic molecules, whereas **1B** is more hydrophilic. The findings of this study demonstrate that comprehensive synthetic investigations of well‐established chemical systems, such as the aluminium hydroxy acetate system, can result in the identification of novel compounds exhibiting unexpected properties thus adding fundamental synthetic and structural knowledge to the fields of inorganic chemistry and porous materials.

## Accession Codes

CCDC‐2361983 and CCDC‐2363306‐2363310 contain the supplementary crystallographic data for this paper. These data can be obtained free of charge via www.ccdc.cam.ac.uk/data_request/cif, or by emailing data_request@ccdc.cam.ac.uk, or by contacting The Cambridge Crystallographic Data Centre, 12 Union Road, Cambridge CB2 1EZ, UK; fax: +44 1223 336033.

## Conflict of Interests

The authors declare no conflict of interest.

1

## Supporting information

As a service to our authors and readers, this journal provides supporting information supplied by the authors. Such materials are peer reviewed and may be re‐organized for online delivery, but are not copy‐edited or typeset. Technical support issues arising from supporting information (other than missing files) should be addressed to the authors.

Supporting Information

## Data Availability

The data that support the findings of this study are available in the supplementary material of this article.
