# Supplementary material for: Unlocking the Chemical and Structural Complexity of Aluminum Hydroxy Acetates: from Commodity Chemicals to Porous Materials
Source: Chemistry. 2024 Nov 22;31(4):e202403634. doi: 10.1002/chem.202403634 (PMC11739832; doi:10.1002/chem.202403634)
Supplement: Supplementary file 1 — Supporting Information [file CHEM-31-e202403634-s001.pdf]

Chemistry–A European Journal

Supporting Information

Unlocking the Chemical and Structural Complexity of Aluminum Hydroxy Acetates: from Commodity Chemicals to Porous Materials

Bastian Achenbach, Lena-Marie Liedtke, Christian Näther, Erik Svensson Grape, A. Ken Inge,* and Norbert Stock*

Supporting Information
©Wiley-VCH 2021
69451 Weinheim, Germany

Unlocking the Chemical and Structural Complexity of Aluminum Hydroxy Acetates: from Commodity Chemicals to Porous Materials

Bastian Achenbach,[a] Lena-Marie Liedtke,[a] Christian Näther,[a] Erik Svensson Grape,[b,c] A. Ken Inge,*[b,d] Norbert Stock*[a,e]

Abstract: Aluminum acetates have been in use for more than a century, but despite their widespread commercial applications, essential scientific knowledge of their synthesis-structure-property relationships is lacking. High-throughput screening, followed by fine tuning and extensive optimization of reaction conditions using Al^{3+}, OH^- and CH_3COO^- ions, has unraveled their complex synthetic chemistry, yielding for the first time the four phase pure products $Al(OH)(O_2CCH_3) \cdot x\ H_2O$ (x = 0, 2) (**1A** and CAU-65, **1B**), $Al_3O(HO_2CCH_3)(O_2CCH_3)_7$ (**2**), and the porous aluminum salt $[Al_{24}(OH)_{56}(CH_3COO)_{12}](OH)_4$ (CAU-55-OH, **3**). Structure determination by electron and X-ray diffraction was carried out and the data suggested porosity for **1B** and **3**, which was confirmed by physisorption experiments. Even the scale-up to the 10 L scale was accomplished for **1A**, **1B** and **3** with yields of up to 1.1 kg (99%). This study of a seemingly simple chemical system provides important information on both fundamental inorganic chemistry and porous materials.

DOI: 10.1002/anie.2024XXXXX

Table of Contents

Table of Contents 2
1. Materials and methods 3
1.1. Chemicals 3
1.2. Methods 3
2. Synthesis 5
2.1. High-throughput investigations 5
2.1.1. High-throughput screening 5
2.1.2. Synthesis optimization of $Al(OH)(O_2CCH_3)_2 \cdot 2\ H_2O$ (**CAU-65, 1B**) 19
2.1.2. Synthesis optimization of $Al_3O(HO_2CCH_3)(O_2CCH_3)_7$ (**2**) 21
2.2. Optimized synthesis conditions and synthesis scale up[12] 24
2.2.1. $Al(OH)(O_2CCH_3)$ (**1A**) 24
2.2.2. CAU-65 (**1B**) 25
2.2.3. $Al_3O(HO_2CCH_3)(O_2CCH_3)_7$ (**2**) 26
2.2.4. CAU-55-OH (**3**) 27
3. Crystal structure determination and refinement 28
3.1. Rietveld, Le Bail plots and crystallographic data 28
3.2. Crystal structure of $Al(OH)(O_2CCH_3)_2$ (**1A**) 33
3.3. Crystal structure of $Al_3(O)(HO_2CCH_3)(O_2CCH_3)_7$ (**2**) 35
3.4. Crystal structure of CAU-55-OH $[Al_{24}(OH)_{56}(O_2CCH_3)_{12}](OH)_4$ (**3**) 36
4. Spectroscopic characterization, elemental analyses and thermal properties 38
4.1. IR spectroscopy 38
4.2. Elemental and EDX analyzes 39
4.3. Thermogravimetric curves 40
4.4. Variable temperature PXRD (VT-PXRD) 42
4.5. Compositions of the compounds 44
5. Sorption properties 45
5.1. Crystallographic data for 1B containing guest molecules 49
6. References 52
Author Contributions 53

1. Materials and methods

1.1. Chemicals

Acetic acid (HO_2CCH_3, VWR International GmbH, > 99 %), acetic acid anhydride ($(CH_3CO)_2O$, thermos scientific, > 99 %), aluminum chloride hexahydrate ($AlCl_3 \cdot 6\,H_2O$, Alfa Aesar GmbH & Co KG, 99 %), aluminum isopropoxide ($Al(OCH(CH_3)_2)_3$, TCI, > 98 %), aluminum sulfate octadecahydrate ($Al_2(SO_4)_3 \cdot 18\,H_2O$, Bernd Kraft GmbH, 99 %), ethanol (C_2H_5OH, Walter-CMP, > 98 %), methanol (CH_3OH, Walter-CMP, > 97 %), sodium acetate trihydrate ($NaO_2CCH_3 \cdot 3\,H_2O$, Grüssing GmbH, 99 %), sodium hydroxide (NaOH, Grüssing GmbH, 99 %), and sodium metaaluminate ($NaAlO_2$, Sigma-Aldrich GmbH, techn. grade) were commercially obtained and used without further purification.

1.2. Methods

HT synthesis optimization

High-throughput studies were performed using our custom-made steel autoclaves[1] with 24 Teflon® inserts with a total volume of 2 mL each. Aqueous solutions of $AlCl_3$ (1.44 mol/L), acetic acid (5.76 mol/L) and NaOH (2 mol/L or 6 mol/L), 5.4 mmol) were mixed, and the reaction volume was kept constant at 1 mL by adding water. After sealing the autoclaves, the reaction vessels were placed in a Memmert UNB 500 oven with forced ventilation and a programmable temperature-time program. The reaction products were separated by centrifugation, washed with water and ethanol and dried at room temperature. Detailed information on the temperature-time programs, the reactions performed and reaction products obtained are given in Section S2.

Synthesis scale up

The molar ratios of the starting materials, the reaction time and the reaction temperature for the synthesis of the title compounds optimized by high-throughput studies were used for the synthesis scale up. The scale-up was carried out increasing the size of the batch reactor step-wise from 1 mL reaction volume to the 10 L scale (scaling factor: 10,000) using 10 mL glass vials as well as 250 mL and 10 L round bottom flasks. Reaction products were separated by centrifugation or filtration, washed with water and ethanol and dried at room temperature.

3D electron diffraction

Three-dimensional electron diffraction data were collected on a JOEL JEM 2100 LaB_6 microscope operating at 200 kV, equipped with a Timepix hybrid pixel detector. The samples were prepared by sprinkling the powder over holey carbon-covered Cu grids. Data collection was carried out using Instamatic.[2] Subsequent data reduction was carried out using XDS[3], and the structures were solved and refined using SHELXT and SHELXS, respectively.[4,5] For low-temperature measurements at 98 K, a Gatan 914 cryo-transfer holder was used.

Single crystal X-ray diffraction (SCXRD)

The single crystal X-ray data for compound **2** were collected on a Rigaku XtaLAB Synergy, Dualflex, HyPix diffractometer unsing Cu-Kα radiation. The structure was solved with ShelXT[5] and by using Olex2[6] as the graphical interface. Structure refinements were performed with version 2016/6 of ShelXL[5] using Least Squares minimization. All non-hydrogen atoms were refined anisotropic. The C-H and O-H H atoms were located in difference map but finally positioned with idealized geometry allowed to rotate but not to tip and were refined isotropic with $U_{iso}(H) = 1.5\,U_{eq}(C, O)$ using a riding model.

CCDC-2361983 contain the supplementary crystallographic data of **2** for this paper. These data can be obtained free charge from the Cambridge Crystallographic Data Centre via http://www.ccdc.cam.ac.uk/data_request/cif.

Powder X-ray diffraction (PXRD)

The PXRD data were collected in transmission geometry using a STOE Stadi MP or a STOE Stadi P-Combi diffractometer equipped both with a MYTHEN 1 K detector and using monochromated Cu-Kα1 radiation. The high-throughput powder X-ray diffraction measurements were performed using a xy-stage. Topas Academic[7,8] was used for indexing and refinements.

CCDC 2363306-2363310 contain the supplementary crystallographic data of **1A** and CAU-65 (**1B-X** with X = activated, MeOH; EtOH, Toluene) for this paper. These data can be obtained free charge from the Cambridge Crystallographic Data Centre via http://www.ccdc.cam.ac.uk/data_request/cif.

Variable temperature powder X-ray diffraction (VT-PXRD)

Variable temperature PXRD (VT-PXRD) was performed using a Stoe capillary furnace. The samples were filled into 0.5 mm quartz capillaries and placed in the capillary furnace. The temperature was varied between 30 °C and the decomposition temperature of the title compounds. The samples were heated in steps of 10 or 20 °C and PXRD data were collected after each heating step.

Elemental and EDX analyses

Energy dispersive spectra were acquired using a Philips ESEM XL30 equipped with an Oxford EDX detector and elemental analyses of the samples was performed using an Elementar Vario MICRO Cube Elemental Analyzer.

IR spectroscopy

IR-spectra of the title compounds were collected using a Bruker ALPHA-FT-IR A220/D-01 with an ATR-unit.

Thermogravimetric measurements

Thermogravimetric measurements were performed on a Linseis STA PT 1000 (airflow = 6 dm^3/h, heating rate = 4 K/min or 8 K/min). The sample amount was approximately 25 mg for each sample.

Sorption measurements

Nitrogen and vapor (water, methanol, ethanol and toluene) sorption measurements were carried out using a BEL Japan Inc. BELSORP-max with nitrogen gas at 77 K and water vapor at 298 K. Prior to the measurements the samples were treated for 4 h at elevated temperatures (70 – 120 °C.) under reduced pressure ($p < 10^{-2}$ mbar). To confirm the integrity and the long-range order of the structures after thermal activation, PXRD patterns of the samples were collected after sorption measurements.

2. Synthesis

2.1. High-throughput investigations

2.1.1. High-throughput screening

The high-throughput investigation starting from $AlCl_3$, CH_3COOH, NaOH and H_2O were carried out using custom-made steel autoclaves[1] with 24 Teflon® inserts with a total volume of 2 mL each (Figure S2.1). Molar ratios were varied between 0.5 – 4, 3 – 14 and 3 – 9 for $AlCl_3$, CH_3COOH and NaOH, respectively. The reactions were carried out at 70, 100 and 130 °C (Figure S2.1, Table S2.2, Table S2.6 – S2.7) and the reaction time was varied between 7 and 33 h (Figure S2.1, Table S2.8 – S2.9). One equivalent corresponds to 0.18 mmol/L. Aqueous solutions of $AlCl_3$ (1.44 mol/L), acetic acid (5.76 mol/L) and NaOH (2 mol/L or 6 mol/L), 5.4 mmol) were mixed, and the reaction volume was kept constant at 1 mL by the addition of water. The reactant solutions were added to the reaction vessels in the following order:

1) $AlCl_3$ (c = 1.44 mol/L)
2) H_2O
3) CH_3COOH (c = 5.76 mol/L)
4) NaOH (c_1 = 2 mol/L, c_2 = 6 mol/L)

After sealing the autoclaves, the reaction vessels were placed in a Memmert UNB 500 oven with forced ventilation and a programmable temperature-time-program (Figure S2.1, right). The reaction products were separated by centrifugation (micro centrifuge Gusto, Heathrow Scientific), washed with water and ethanol and dried at room temperature.

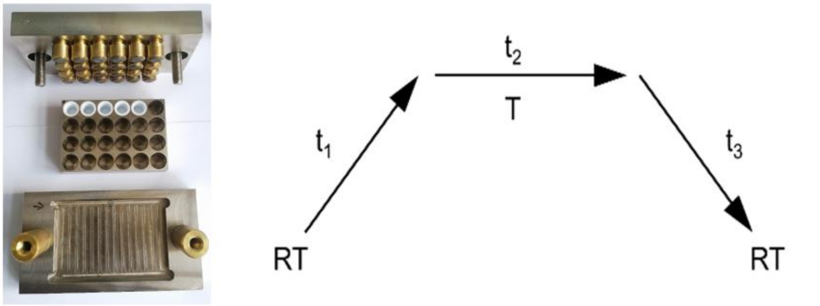

Figure S2.1. Left: Custom-made steel autoclaves[1] with up to 24 Teflon® inserts with a total volume of 2 mL each. **Right**: General temperature-time program used for the high-throughput investigations. The HT-autoclaves were heated up to a specific temperature (T) over 1 h (t_1), held at this temperature for 4, 12 or 30 h (t_2) and cooled down to room temperature over 2 h (t_3).

Table S2.1. List of starting materials employed in the systematic investigation of the chemical system $AlCl_3$ / CH_3COOH / NaOH at constant $AlCl_3$ concentrations (**0.5 eq.**). The reaction temperature was set to 100 °C and the heating program was set to 1-12-2h (t_1-t_2-t_3).

	molar ratios			$V_{solution}$ (µL)				T (°C); t_1-t_2-t_3 (h)	Obtained crystalline phases
	$AlCl_3$	CH_3COOH	NaOH	$AlCl_3$	CH_3COOH	NaOH	H_2O		
A1		4			125		453		
A2		5			156		421		
A3		6	4		188	360[a]	390		
A4		7			219		359		
A5		8			250		265		
A6		9			281		234		
A7		4			125		363		
A8		5			156		331		
A9		6			188		300		
A10		7	5		219	450[a]	269		
A11		8			250		238		
A12		10			313		175		
A13		12			375		113		
A14		14			438		50		
A15		4			125		273		
A16		5			156		241		
A17	0.5	6		62.5	188	540[a]	210	100 °C; 1-12-2 h	
A18		7	6		219		179		
A19		8			250		85		
A20		10			313		23		
A21		12			375	180[b]	320		
A22		14			438		258		
A23		4			125		183		
A24		5			156	630[a]	151		
A25		6			188		120		
A26		7	7		219		89		
A27		8			250		383		
A28		10			313	210[b]	320		
A29		12			375		290		
A30		14			438		228		
A31		4			125	720[a]	93		
A32		8	8		250	240[b]	448		
A33		10			313		385		

A34	12		375		323		
A35	14		438		260		
A36	4	9	125	810[a]	4		

[a] c_1 = 2 mol/L, [b] c_2 = 6 mol/L

Table S2.2. List of starting materials employed in the systematic investigation of the chemical system $AlCl_3$ / CH_3COOH / NaOH at constant $AlCl_3$ concentrations (**1 eq.**). The reaction temperature was set to 100 °C and the heating program was set to 1-12-2h (t_1-t_2-t_3).

	molar ratios			$V_{solution}$ (µL)				T (°C); t_1-t_2-t_3 (h)	Obtained crystalline phases
	$AlCl_3$	CH_3COOH	NaOH	$AlCl_3$	CH_3COOH	NaOH	H_2O		
B1		3			94		421		
B2		4			125		390		
B3		5			156		359		
B4		6	4		188	360[a]	328		
B5		7			219		296		
B6		8			250		265		
B7		9			281		234		
B9		4			125		300		
B10		5			156		269		
B11		6			188		238		
B12		7	5		219		206		
B13		8			250		175		
B14		10			313		113		
B15		12			375		50		
B16		14			438	150[b]	288		
B17		4			125		210		
B18	1	5		125	156		179	100 °C; 1-12-2 h	
B19		6			188	540[a]	148		
B20		7	6		219		116		
B21		8			250		85		
B22		10			313		23		
B23		12			375	180[b]	320		
B24		14			438		258		
B25		4			125		120		
B26		5			156	630[a]	89		
B27		6			188		58		
B28		7	7		219		26		
B29		8			250		415		
B30		10			313	210[b]	353		
B31		12			375		290		
B32		14			438		228		
B33		4			125	720[a]	30		
B34		8	8		250	240[b]	385		
B35		10			313		323		

B36	12		375		260		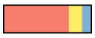
B37	14		438		198		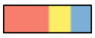
B38	4	9	125	270[b]	480		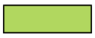

[a] c_1 = 2 mol/L, [b] c_2 = 6 mol/L

Table S2.3. List of starting materials employed in the systematic investigation of the chemical system $AlCl_3$ / CH_3COOH / NaOH at constant $AlCl_3$ concentrations (**2 eq.**). The reaction temperature was set to 100 °C and the heating program was set to 1-12-2h (t_1-t_2-t_3).

	molar ratios			$V_{solution}$ (µL)				T (°C); t_1-t_2-t_3 (h)	Obtained crystalline phases
	$AlCl_3$	CH_3COOH	NaOH	$AlCl_3$	CH_3COOH	NaOH	H_2O		
C1	2	4	4	250	125	360[a]	265	100 °C; 1-12-2 h	
C2		5			156		234		
C3		6			188		203		
C4		7			219		171		
C5		8			250		140		
C6		9			281		109		
C7		4	5		125	450[a]	175		
C8		5			156		144		
C9		6			188		113		
C10		7			219		80		
C11		8			250		50		
C12		10			313	150[b]	288		
C13		12			375		225		
C14		14			438		163		
C15		4	6		125	540[a]	85		
C16		5			156		54		
C17		6			188		23		
C18		7			219	180[b]	351		
C19		8			250		320		
C20		10			313		258		
C21		12			375		195		
C22		14			438		133		
C23		4	7		125	210[b]	415		
C24		5			156		384		
C25		6			188		353		
C26		7			219		321		
C27		8			250		290		
C28		10			313		228		
C29		12			375		165		
C30		14			438		103		
C31		4	8		125	240	385		
C32		8			250		260		
C33		10			313		198		
C34		12			375		135		

C35		14			438		73		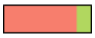
C36		4	9		125	270	355		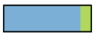

[a] c_1 = 2 mol/L, [b] c_2 = 6 mol/L

SUPPORTING INFORMATION

Table S2.4. List of starting materials employed in the systematic investigation of the chemical system $AlCl_3$ / CH_3COOH / NaOH at constant $AlCl_3$ concentrations (**3 eq.**). The reaction temperature was set to 100 °C and the heating program was set to 1-12-2h (t_1-t_2-t_3).

	molar ratios			$V_{solution}$ (µL)				T (°C); t_1-t_2-t_3 (h)	Obtained crystalline phases
	$AlCl_3$	CH_3COOH	NaOH	$AlCl_3$	CH_3COOH	NaOH	H_2O		
D1	3	4	4	375	125	360[a]	140	100 °C; 1-12-2 h	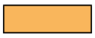
D2		5			156		109		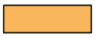
D3		6			188		78		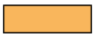
D4		7			219		46		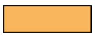
D5		4	5		125	450[a]	50		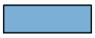
D6		5			156		19		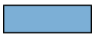
D7		6			188	150[b]	288		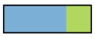
D8		7			219		256		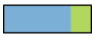
D9		4	6		125	180[b]	320		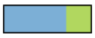
D10		5			156		289		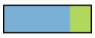
D11		6			188		258		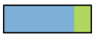
D12		7			219		226		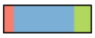
D13		4	7		125	210[b]	290		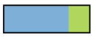
D14		5			156		259		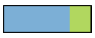
D15		6			188		228		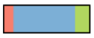
D16		7			219		196		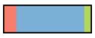

[a] c_1 = 2 mol/L, [b] c_2 = 6 mol/L

Table S2.5. List of starting materials employed in the systematic investigation of the chemical system $AlCl_3$ / CH_3COOH / NaOH at constant $AlCl_3$ concentrations (**4 eq.**). The reaction temperature was set to 100 °C and the heating program was set to 1-12-2h (t_1-t_2-t_3).

	molar ratios			$V_{solution}$ (µL)				T (°C); t_1-t_2-t_3 (h)	Obtained crystalline phases
	$AlCl_3$	CH_3COOH	NaOH	$AlCl_3$	CH_3COOH	NaOH	H_2O		
E1	4	4	4	500	125	120[b]	255	100 °C; 1-12-2 h	
E2		5			156		224		
E3		6			188		193		
E4		7			219		161		
E5		4	5		125	150[b]	225		
E6		5			156		194		
E7		6			188		163		
E8		7			219		131		
E9		4	6		125	180[b]	195		
E10		5			156		164		
E11		6			188		133		
E12		7			219		101		
E13		4	7		125	210[b]	165		
E14		5			156		134		
E15		6			188		103		
E16		7			219		71		

[a] c_1 = 2 mol/L, [b] c_2 = 6 mol/L

Table S2.6. List of starting materials employed in the systematic investigation of the chemical system $AlCl_3$ / CH_3COOH / NaOH at constant $AlCl_3$ concentrations (1 eq.). The reaction temperature was set to **70 °C** and the heating program was set to 1-12-2h (t_1-t_2-t_3).

	molar ratios			$V_{solution}$ (µL)				T (°C); t_1-t_2-t_3 (h)	Obtained crystalline phases
	$AlCl_3$	CH_3COOH	NaOH	$AlCl_3$	CH_3COOH	NaOH	H_2O		
F1	1	4	4	125	125	360[a]	390	70 °C; 1-12-2 h	
F2		5			156		259		
F3		6			188		328		
F4		7			219		296		
F5		4	5		125	450[a]	300		
F6		5			156		269		
F7		6			188		238		
F8		7			219		206		
F9		8			250		175		
F10		10			313		113		
F11		12			375		50		
F12		14			438	150[b]	288		
F13		4	6		125	540[a]	210		
F14		5			156		179		
F15		6			188		148		
F16		7			219		116		
F17		8			250		85		
F18		10			313		23		
F19		12			375	180[b]	320		
F20		14			438		258		
F21		4	7		125	630[a]	120		
F22		5			156		89		
F23		6			188		58		
F24		7			219		26		
F25		8			250	210[b]	415		
F26		10			313		353		
F27		12			375		290		
F28		14			438		228		
F29		8	8		250	240[b]	385		
F30		10			313		323		
F31		12			375		260		
F32		14			438		198		

[a] c_1 = 2 mol/L, [b] c_2 = 6 mol/L

Table S2.7. List of starting materials employed in the systematic investigation of the chemical system $AlCl_3$ / CH_3COOH / NaOH at constant $AlCl_3$ concentrations (1 eq.). The reaction temperature was set to **130 °C** and the heating program was set to 1-12-2h (t_1-t_2-t_3).

	molar ratios			$V_{solution}$ (µL)				T (°C); t_1-t_2-t_3 (h)	Obtained crystalline phases
	$AlCl_3$	CH_3COOH	NaOH	$AlCl_3$	CH_3COOH	NaOH	H_2O		
G1		4			125		390		
G2		5	4		156	360[a]	259		
G3		6			188		328		
G4		7			219		296		
G5		4			125		300		
G6		5	5		156		269		
G7		6			188		238		
G8		7			219	450[a]	206		
G9		8			250		175		
G10		10			313		113		
G11		12			375		50		
G12		14			438	150[b]	288		
G13		4			125		210		
G14		5			156		179		
G15		6			188	540[a]	148		
G16	1	7	6	125	219		116	130 °C; 1-12-2 h	
G17		8			250		85		
G18		10			313		23		
G19		12			375	180[b]	320		
G20		14			438		258		
G21		4			125		120		
G22		5			156	630[a]	89		
G23		6			188		58		
G24		7	7		219		26		
G25		8			250		415		
G26		10			313	210[b]	353		
G27		12			375		290		
G28		14			438		228		
G29		8			250		385		
G30		10	8		313	240[b]	323		
G31		12			375		260		
G32		14			438		198		

[a] c_1 = 2 mol/L, [b] c_2 = 6 mol/L

Table S2.8. List of starting materials employed in the systematic investigation of the chemical system $AlCl_3$ / CH_3COOH / NaOH at constant $AlCl_3$ concentrations (1 eq.). The reaction temperature was set to 100 °C and the heating program was set to **1-4-2h (t_1-t_2-t_3)**.

	molar ratios			$V_{solution}$ (µL)				T (°C); t_1-t_2-t_3 (h)	Obtained crystalline phases
	$AlCl_3$	CH_3COOH	NaOH	$AlCl_3$	CH_3COOH	NaOH	H_2O		
H1		4			125		390		
H2		5	4		156	360[a]	259		
H3		6			188		328		
H4		7			219		296		
H5		4			125		300		
H6		5			156		269		
H7		6			188		238		
H8		7	5		219	450[a]	206		
H9		8			250		175		
H10		10			313		113		
H11		12			375		50		
H12		14			438	150[b]	288		
H13		4			125		210		
H14		5			156		179		
H15		6			188	540[a]	148		
H16	1	7	6	125	219		116	100 °C; 1-4-2 h	
H17		8			250		85		
H18		10			313		23		
H19		12			375	180[b]	320		
H20		14			438		258		
H21		4			125		120		
H22		5			156	630[a]	89		
H23		6			188		58		
H24		7	7		219		26		
H25		8			250		415		
H26		10			313	210[b]	353		
H27		12			375		290		
H28		14			438		228		
H29		8			250		385		
H30		10	8		313	240[b]	323		
H31		12			375		260		
H32		14			438		198		

[a] c_1 = 2 mol/L, [b] c_2 = 6 mol/L

Table S2.9. List of starting materials employed in the systematic investigation of the chemical system $AlCl_3$ / CH_3COOH / NaOH at constant $AlCl_3$ concentrations (1 eq.). The reaction temperature was set to 100 °C and the heating program was set to **1-30-2h (t_1-t_2-t_3)**.

	molar ratios			$V_{solution}$ (µL)				T (°C); t_1-t_2-t_3 (h)	Obtained crystalline phases
	$AlCl_3$	CH_3COOH	NaOH	$AlCl_3$	CH_3COOH	NaOH	H_2O		
I1		4			125		390		
I2		5	4		156	360[a]	259		
I3		6			188		328		
I4		7			219		296		
I5		4			125		300		
I6		5	5		156		269		
I7		6			188		238		
I8		7			219	450[a]	206		
I9		8			250		175		
I10		10			313		113		
I11		12			375		50		
I12		14			438	150[b]	288		
I13		4			125		210		
I14		5			156		179		
I15		6			188	540[a]	148		
I16	1	7	6	125	219		116	100 °C; 1-30-2 h	
I17		8			250		85		
I18		10			313		23		
I19		12			375	180[b]	320		
I20		14			438		258		
I21		4			125		120		
I22		5			156	630[a]	89		
I23		6			188		58		
I24		7	7		219		26		
I25		8			250		415		
I26		10			313	210[b]	353		
I27		12			375		290		
I28		14			438		228		
I29		8			250		385		
I30		10	8		313	240[b]	323		
I31		12			375		260		
I32		14			438		198		

[a] c_1 = 2 mol/L, [b] c_2 = 6 mol/L

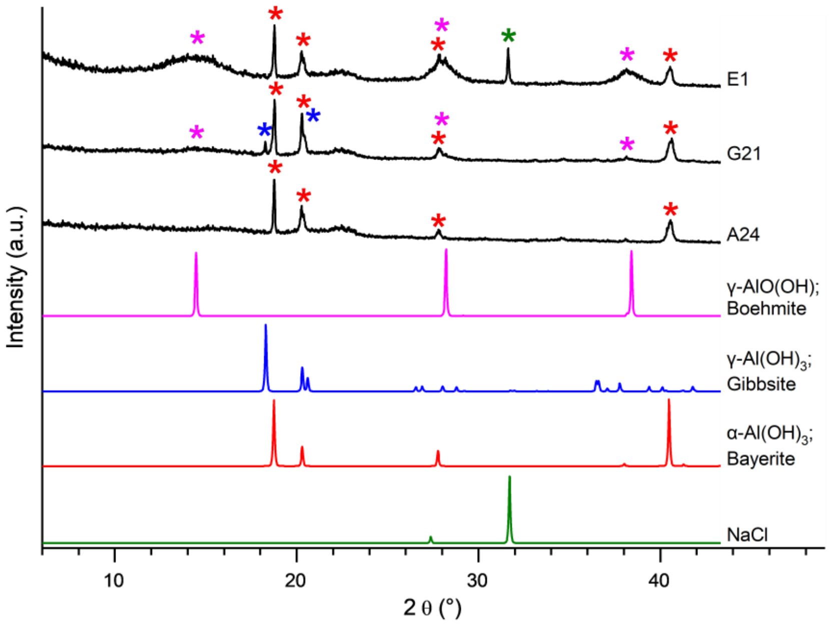

Figure S2.2. PXRD data of different polymorphs of $AlO_x(OH)_y$ (Boehmite (ICSD: 27865), Gibbsite (ICSD: 36233) and Bayerite (ICSD: 38108)[9] obtained during the systematic high-throughput investigation of the system Al^{3+} / NaOH / CH_3COOH / H_2O. For some reactions residues of NaCl (ICSD: 52232) were identified.

2.1.2. Synthesis optimization of $Al(OH)(O_2CCH_3)_2 \cdot 2\,H_2O$ (CAU-65, 1B)

To optimize the synthesis conditions of the pseudopolymorphs **1B**, the initial results of our HT screening (Section S2.1) were used in further optimization reactions employing different aluminum salts ($AlCl_3$, $Al_2(SO_4)_3$ and $Al(NO_3)_3$) (Figure S2.3), reaction temperatures (25 °C < T < 100 °C) and reaction times (24 h < t < 96 h) (Figure S2.4). The molar ratio aluminum salt ($AlCl_3$, $Al(NO_3)_3$, $Al_2(SO_4)_3$) to acetic acid to sodium hydroxide was fixed to 3 : 12 : 10 for all reactions since at these ratios the most crystalline product was found in the initial HT screening. The reactions were carried out using a *Biometra-Tgradient* temperature gradient oven and 500 µL Eppendorf© vials with a reaction volume of 100 µL. Some of the results are presented in this section as crystallization diagrams (Figure S2.3 and Figure S2.4) and general trends that were identified taking into account all syntheses performed are described below:

The two pseudopolymorphs **1A** and **1B** were formed over a wide range of reaction temperatures (25 °C < T < 150°C) and reaction times (5 min < t < 7 d) using different aluminum salts ($AlCl_3$, $Al_2(SO_4)_3$ and $Al(NO_3)_3$) and various molar ratios of aluminum salt, acetic acid and sodium hydroxide. The use of $Al_2(SO_4)_3$ as the aluminum source was the most versatile and allowed the formation of the pseudopolymorphs over a wide range of reaction temperatures und times.
$Al(OH)(O_2CCH_3)_2$ (**1A**) was formed in high yields preferentially at high reaction temperatures (60 °C < T < 150 °C) and high concentrations of acetic acid and low concentrations of sodium hydroxide (low pH values). A combination of low reaction temperatures (25 °C < T < 60°C), long reaction times (t ≥ 20 h) and high aluminum concentrations led to the formation of phase-pure $Al(OH)(O_2CCH_3)_2 \cdot 2\,H_2O$ (**1B**), whereas higher reactions temperatures resulted in phase mixtures of **1A** and **1B**.
While long reaction times, low aluminum concentrations and low NaOH concentrations led to the formation of highly crystalline products, the use of high aluminum and NaOH concentrations led to high yields and products of lower crystallinity.

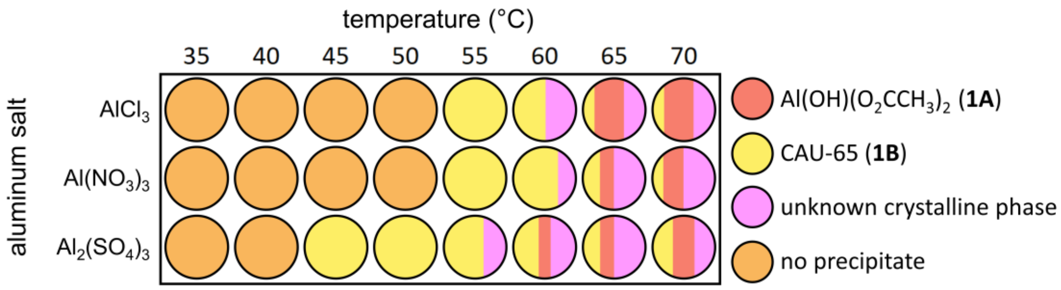

Figure S2.3. Crystallization diagram of crystalline phases observed in the chemical system consisting of an aluminum salt ($AlCl_3$, $Al(NO_3)_3$, $Al_2(SO_4)_3$), acetic acid and sodium hydroxide with a molar ratio of 3 : 12 : 10 for reactions carried out in a temperature gradient oven (35 °C ≤ T ≤ 70°C) at four different reaction times (24, 48, 72 and 96 h). The observed phases are color-coded and the pie charts visualize the proportions of the respective crystalline phases.

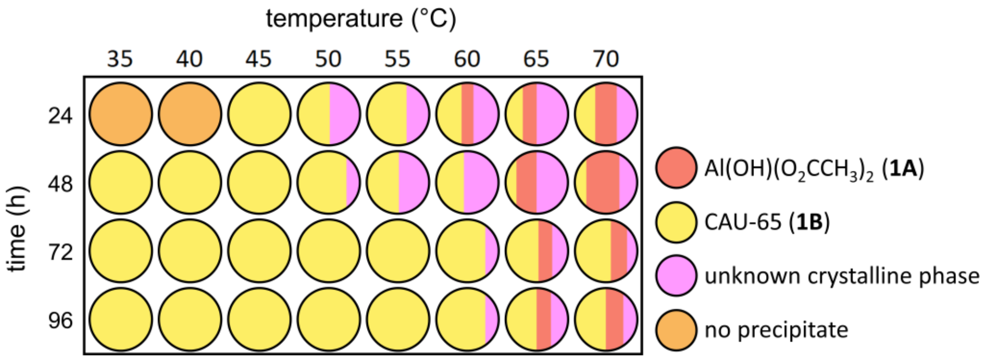

Figure S2.4. Crystallization diagram of crystalline phases observed in the chemical system $Al_2(SO_4)_3$ / CH_3COOH / NaOH / H_2O with a molar ratio of 3 : 12 : 10 for reactions carried out in a temperature gradient oven (35 °C ≤ T ≤ 70°C) and using four different reaction times (24, 48, 72 and 96 h). The observed phases are color-coded and the pie charts visualize the proportions of the respective crystalline phases.

Table S2.10. List of starting materials used to optimize the synthesis of the two pseudopolymorphs of $Al(OH)(O_2CCH_3)_2 \cdot x\ H_2O$ (x = 0, 2) (**1A** and **1B**). Three different aluminum salts, eight different reaction temperatures and four different reaction times were used. For each synthesis a stock solution of 6 mL was prepared for each aluminum salt, and 100 µL was transferred into each Eppendorf© tube and placed in the temperature gradient oven. One equivalent corresponds to a concentration of 1.44 mmol/L.

<table>
<tr><th rowspan="2">Aluminum salt</th><th rowspan="2">$V_{solution}$ (mL)</th><th colspan="4">V (mL); [equivalents]</th><th rowspan="2">T (°C)</th><th rowspan="2">T (h)</th></tr>
<tr><th>Aluminum salt solution</th><th>CH_3COOH</th><th>NaO_2CCH_3</th><th>H_2O</th></tr>
<tr><td>$AlCl_3 \cdot 6\ H_2O$</td><td rowspan="3">6 mL</td><td rowspan="3">2.25 mL[a],
[3]</td><td rowspan="3">0.375 mL[b],
[2]</td><td rowspan="3">2.5 mL[c],
[10]</td><td rowspan="3">3.13 mL</td><td rowspan="3">35 - 70
(ΔT = 5 °C)</td><td rowspan="2">24 h</td></tr>
<tr><td>$Al(NO_3)_3 \cdot 9\ H_2O$</td></tr>
<tr><td>$Al_2(SO_4)_3 \cdot 18\ H_2O$</td><td>24 - 96 h
(Δt = 24 h)</td></tr>
</table>

[a] c_{Al} = 1.44 mol/L; [b] diluted acetic acid, c = 5.76 mol/L; [c] sodium acetate solution, c = 4.32 mol/L

2.1.3. Synthesis optimization of $Al_3O(HO_2CCH_3)(O_2CCH_3)_7$ (2)

Reproduction of literature known syntheses for $Al(O_2CCH_3)_3$

(1) Naegeli *et al*.:[10] The aluminum acetate $Al_3O(HO_2CCH_3)(O_2CCH_3)_7$ (**2**) was initially obtained by following a synthesis procedure published by Naegeli *et al*. in 1938 (Figure 2.5).[10] For the synthesis, 10 g (371 mmol) aluminum powder, 192 mL (3.35 mol) glacial acetic acid and 9.3 mL (98 mmol) acetic anhydride were added to a 250 mL round bottom flask. The reaction mixture was heated under reflux conditions for 4.5 h. The reaction product was separated by filtration and dried at room temperature.

(2) Pande *et al*.:[11] The aluminum acetate $Al_3O(HO_2CCH_3)(O_2CCH_3)_7$ (**2**) was also obtained by following a synthesis procedure published by Pande *et al*. in 1956[11] (Figure 2.5). For the synthesis, 3 g (14.6 mmol) aluminum isopropoxide ($Al(O\text{-}iPr)_3$) and 20 mL (21.2 mmol) acetic anhydride (Ac_2O) were placed in a 100 mL round bottom flask. The reaction mixture was heated under reflux conditions for 4.5 h. The reaction product was separated by filtration and dried at room temperature.

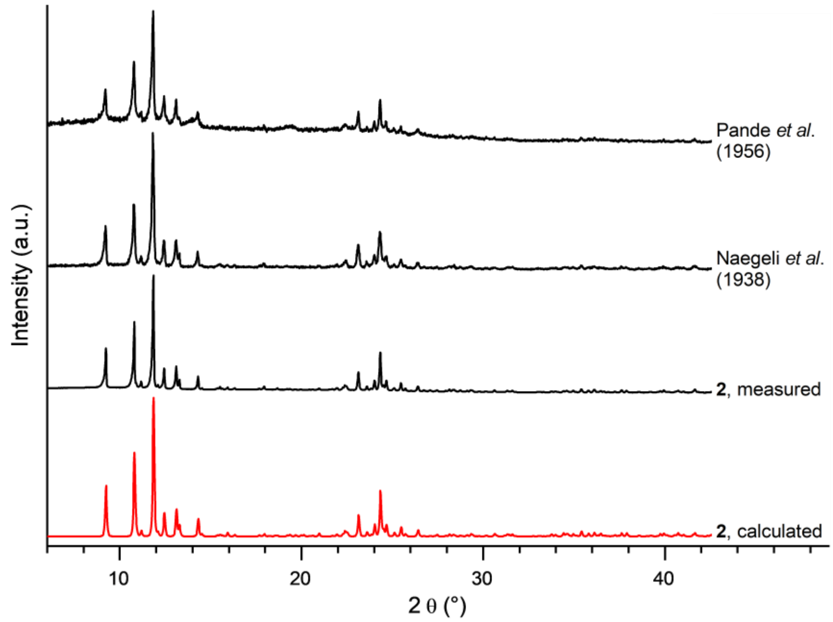

Figure 2.5: Powder X-ray diffraction data of **2** synthesized using literature known synthesis procedures for $Al(O_2CCH_3)_3$.[10,11] The measured and calculated x-ray diffraction pattern of **2** are shown.

Synthesis optimization

For the synthesis optimization of $Al_3O(HO_2CCH_3)(O_2CCH_3)_7$ (**2**) the influence of the molar ratios of aluminum isopropoxide ($Al(O\text{-}iPr)_3$), glacial acetic acid (AcOH) and acetic anhydride (Ac_2O) were investigated using the HT method. For the syntheses a total reaction volume of 800 µL was used for each reaction carried out under solvothermal synthesis conditions at a reaction temperature of 140 °C. Two molar amounts of the aluminum source were used while varying the ratio between glacial acetic acid and acetic anhydride (Table 2.11). Low concentrations of acetic anhydride led to the formation of **1A** while high amounts of acetic anhydride resulted in the formation of **2**, but with a lower crystallinity. The use of 35 mg $Al(O\text{-}iPr)_3$ 600 µL AcOH, 200 µL Ac_2O led to the formation of single crystals of **2**.

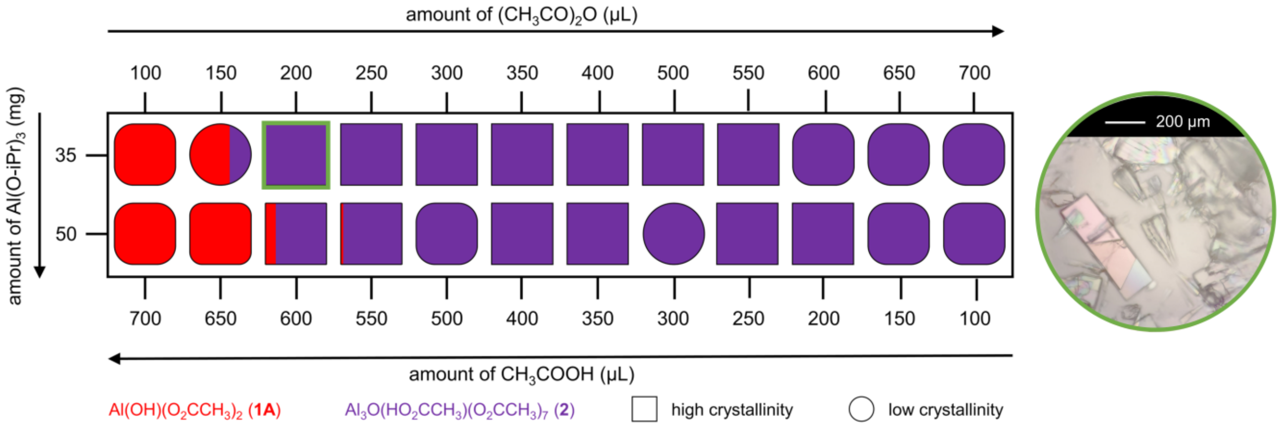

Figure S2.6: Crystallization diagram obtained investigating the influence of the molar ratios of the starting materials ($Al(O\text{-}iPr)_3$, glacial acetic acid (AcOH) and acetic anhydride (Ac_2O)). Each color corresponds to a distinct crystalline phase. Single crystals (highlighted in green) were obtained using 35 mg $Al(O\text{-}iPr)_3$, 200 µL Ac_2O and 600 µL AcOH.

Table S2.11: List of starting materials used for the synthesis optimization of $Al_3O(HO_2CCH_3)(O_2CCH_3)_7$ (**2**). All reactions were carried out using the HT method with a total reaction volume of 800 µL for each Teflon reactor. All products were obtained using aluminum isopropoxide as the aluminum source, a reaction temperature of 140 °C, and a reaction time-program of 3-24-3 h.

**V**solution (µL)	**V (µL); [n]**			**T** (°C)	**t** (h)
	**Aluminum salt**	**CH_3COOH**	**$(CH_3CO)_2O$**		
800	35 mg, [171 µmol]	700 µL, [12.23 mmol]	100 µL, [1.06 mmol]	140	3-24-3
	35 mg, [171 µmol]	650 µL, [11.36 mmol]	150 µL, [1.59 mmol]		
	35 mg, [171 µmol]	600 µL, [10.48 mmol]	200 µL, [2.12 mmol]		
	35 mg, [171 µmol]	550 µL, [9.61 mmol]	250 µL, [2.64 mmol]		
	35 mg, [171 µmol]	500 µL, [8.74 mmol]	300 µL, [3.17 mmol]		
	35 mg, [171 µmol]	450 µL, [7.86 mmol]	350 µL, [3.70 mmol]		
	35 mg, [171 µmol]	100 µL, [1.75 mmol]	700 µL, [7.41 mmol]		
	35 mg, [171 µmol]	150 µL, [2.62 mmol]	650 µL, [6.88 mmol]		
	35 mg, [171 µmol]	200 µL, [3.49 mmol]	600 µL, [6.35 mmol]		
	35 mg, [171 µmol]	250 µL, [4.37 mmol]	550 µL, [5.82 mmol]		
	35 mg, [171 µmol]	300 µL, [5.24 mmol]	500 µL, [5.29 mmol]		
	35 mg, [171 µmol]	400 µL, [6.99 mmol]	400 µL, [4.23 mmol]		
	50 mg, [245 µmol]	700 µL, [12.23 mmol]	100 µL, [1.06 mmol]		
	50 mg, [245 µmol]	650 µL, [11.36 mmol]	150 µL, [1.59 mmol]		
	50 mg, [245 µmol]	600 µL, [10.48 mmol]	200 µL, [2.12 mmol]		
	50 mg, [245 µmol]	550 µL, [9.61 mmol]	250 µL, [2.64 mmol]		
	50 mg, [245 µmol]	500 µL, [8.74 mmol]	300 µL, [3.17 mmol]		
	50 mg, [245 µmol]	450 µL, [7.86 mmol]	350 µL, [3.70 mmol]		
	50 mg, [245 µmol]	100 µL, [1.75 mmol]	700 µL, [7.41 mmol]		
	50 mg, [245 µmol]	150 µL, [2.62 mmol]	650 µL, [6.88 mmol]		
	50 mg, [245 µmol]	200 µL, [3.49 mmol]	600 µL, [6.35 mmol]		
	50 mg, [245 µmol]	250 µL, [4.37 mmol]	550 µL, [5.82 mmol]		
	50 mg, [245 µmol]	300 µL, [5.24 mmol]	500 µL, [5.29 mmol]		
	50 mg, [245 µmol]	400 µL, [6.99 mmol]	400 µL, [4.23 mmol]		

2.2. Optimized synthesis conditions and synthesis scale up[12]

In the following sections (Section 2.2.1 – 2.2.4), the optimized synthesis conditions for $Al(OH)(O_2CCH_3)$ (**1A**), $Al(OH)(O_2CCH_3) \cdot 2\ H_2O$ (**1B**), $Al_3O(HO_2CCH_3)(O_2CCH_3)_7$ (**2**) and CAU-55-OH (**3**) used for the synthesis scale up are described.

2.2.3. $Al(OH)(O_2CCH_3)$ (1A)

The synthesis of $Al(OH)(O_2CCH_3)$ (**1A**) in high crystallinity (Table S2.12-2) for Rietveld refinement was performed in our custom-made steel autoclaves with 24 Teflon® inserts with a total volume of 2 mL. For the synthesis scale up, the optimized synthesis conditions for $Al(OH)(O_2CCH_3)$ (**1A**) leading to high yields (Table S2.12-1) were used.

Synthesis of 1A in a 10 mL Pyrex glass vial (Table S2.12-3)
In a 10 mL Duran® glass vial, 0.4 mL of water and 1.00 mL of an aqueous solution of $Al_2(SO_4)_3$ (c_{Al} = 1.44 mol/L, 0.72 mmol), were mixed with 3 mL (5.76 mol/L, 17.3 mmol) of dilute acetic acid and 3.6 mL of diluted NaOH (2 mol/L, 7.2 mmol). Subsequently the reaction vial was sealed and the reaction mixture was heated at 100 °C for 20 h under stirring using a heating plate with magnetic stirrer (MR Hei-Tec, Heidolph). After cooling to room temperature, the reaction product was separated by centrifugation (*Allegra 64R, Beckman Coulter)*, washed with water and ethanol and dried at room temperature to yield 242 mg (1.43 mmol, 99% based on Al^{3+}, calculated using the composition $Al(OH)(O_2CCH_3)_2 \cdot 0.4\ H_2O$).

Synthesis of 1A in a 250 mL round bottom flask (Table S2.12-4)
In a 250 mL round bottom flask, 9.6 g (14.4 mmol) of $Al_2(SO_4)_3 \cdot 18\ H_2O$ were dissolved in 68.2 mL of water and a mixture of 19.8 mL (346 mmol) glacial acetic acid and 72 mL of diluted sodium hydroxide solution (2 mol/L, 144 mmol) was added. The reaction mixture was heated and stirred under reflux conditions for 20 h using a heating plate with magnetic stirrer (MR Hei-Tec, Heidolph). After cooling to room temperature, the reaction product was separated by filtration and washed with water and ethanol to yield 242 mg (1.43 mmol, 99% based on Al^{3+}, calculated using the composition $Al(OH)(O_2CCH_3)_2 \cdot 0.4\ H_2O$).

Synthesis of 1A in a 10 L round bottom flask (Table S2.12-5)
In a 10 L round bottom flask, 600 g (0.9 mol) of $Al_2(SO_4)_3 \cdot 18\ H_2O$ were dissolved in 4.26 L of water and a mixture of 1.24 L (21.6 mol) glacial acetic acid and 4.5 L of diluted sodium hydroxide solution (2 mol/L, 9 mol) was added. The reaction mixture was heated and stirred under reflux conditions for 20 h. After cooling to room temperature, the reaction product was separated by filtration and washed with water and ethanol to 303 g (1.83 mol, 99% based on Al^{3+}, calculated using the composition $Al(OH)(O_2CCH_3)_2 \cdot 0.4\ H_2O$).

Table S2.12. List of reactor types and starting materials used for the synthesis scale-up of $Al(OH)(O_2CCH_3)$ (**1A**) by gradually increasing the size of the batch reactor. One equivalent corresponds to a concentration of 1.44 mmol/L. The label L corresponds to the scale-up syntheses and B to the high-throughput reaction.

No.	Type of reactor	$V_{solution}$	V (mL); m (g), [equivalents]				T; t
			$Al_2(SO_4)_3$	CH_3COOH	NaOH	H_2O	
1	Multiclave with 2 mL Teflon inserts	1 mL	0.125 mL[a], [0.5]	0.375 mL[c], [12]	0.45 mL[e], [5]	0.05 mL	100 °C; 12 h
2		1 mL	0.125 mL[a], [0.5]	0.375 mL[c], [12]	0.18 mL[e], [2]	0.32 mL	
3	Pyrex glass tubes	8 mL	1.0 mL[a], [0.5]	3.0 mL[c], [12]	3.6 mL[e], [5]	0.4 mL	100 °C; 20 h
4	Round bottom flask with stirrer	160 mL	9.6 g[b], [0.5]	19.8 mL[d], [12]	72.0 mL[e], [5]	68.2 mL	
5		10000 mL	600 g[b], [0.5]	1240 mL[d], [12]	4500 mL[e], [5]	4260 mL	

[a] c_{Al} = 1.44 mol/L; [b] $Al_2(SO_4)_3 \cdot 18\ H_2O$; [c] diluted acetic acid, c = 5.76 mol/L; [d] glacial acetic acid, c = 17.5 mol/L; [e] c_{NaOH} = 2.0 mol/L

2.2.4. CAU-65 (1B)

The molar ratios of the starting materials for the synthesis of CAU-65 (**1B**) optimized by high-throughput investigations (Table S2.13-1) were used for the synthesis scale up (Table S2.13-(2-4).)

Synthesis of 1B in a 10 mL Pyrex glass vial (Table S2.13-2)
In a 10 mL Duran® glass vial, 0.4 mL of water and 1.00 mL of an aqueous solution of $Al_2(SO_4)_3$ (c_{Al} = 1.44 mol/L, 0.72 mmol), were mixed with 3 mL (5.76 mol/L, 17.3 mmol) of dilute acetic acid and 3.6 mL of dilute NaOH (2 mol/L, 7.2 mmol). Subsequently the reaction vial was sealed and the reaction mixture was heated at 100 °C for 20 h under stirring using a heating plate with magnetic stirrer (MR Hei-Tec, Heidolph). After cooling to room temperature, the reaction product was separated by centrifugation (*Allegra 64R, Beckman Coulter)*, washed with water and ethanol and dried at room temperature to yield 910 mg (4.28 mmol, 99% based on Al^{3+}, calculated using the composition $Al(OH)(O_2CCH_3)_2 \cdot 2.8\ H_2O$).

Synthesis of 1B in a 250 mL round bottom flask (Table S2.13-3)
In a 250 mL round bottom flask, 9.6 g (14.4 mmol) of $Al_2(SO_4)_3 \cdot 18\ H_2O$ were dissolved in 68.2 mL of water and a mixture of 19.8 mL (346 mmol) glacial acetic acid and 72 mL of diluted sodium hydroxide solution (2 mol/L, 144 mmol) was added. The reaction mixture was heated and stirred under reflux conditions for 20 h using a heating plate with magnetic stirrer (MR Hei-Tec, Heidolph). After cooling to room temperature, the reaction product was separated by filtration and washed with water and ethanol to yield 18.3 g (86.3 mmol, 97% based on Al^{3+}, calculated using the composition $Al(OH)(O_2CCH_3)_2 \cdot 2.8\ H_2O$).

Synthesis of 1B in a 10 L round bottom flask (Table S2.13-4)
In a 10 L round bottom flask, 600 g (0.9 mol) of $Al_2(SO_4)_3 \cdot 18\ H_2O$ were dissolved in 4.26 L of water and a mixture of 1.24 L (21.6 mol) glacial acetic acid and 4.5 L of diluted sodium hydroxide solution (2 mol/L, 9 mol) was added. The reaction mixture was heated and stirred under reflux conditions for 20 h. After cooling to room temperature, the reaction product was separated by filtration and washed with water and ethanol to yield 1097 g (5.16 mol, 96% based on Al^{3+}, calculated using the composition $Al(OH)(O_2CCH_3)_2 \cdot 2.8\ H_2O$).

Table S2.13. List of reactor types and starting materials used for the synthesis scale-up of CAU-65 (**1B**) by gradually increasing the size of the batch reactor. One equivalent corresponds to a concentration of 1.44 mmol/L.

No.	Type of reactor	$V_{solution}$	V (mL); m (g), [equivalents]				T; t
			$Al_2(SO_4)_3$	CH_3COOH	NaO_2CCH_3	H_2O	
1	Multiclave with 2 mL Teflon inserts	1 mL	0.375 mL[a], [1.5]	0.063 mL[c], [2]	0.417 mL[e], [10]	0.146 mL	50 °C; 12 h
2	Pyrex glass tubes	8 mL	3.00 mL[a], [1.5]	0.50 mL[c], [2]	3.33 mL[e], [10]	1.17 mL	50 °C; 20 h
3	Round bottom flask with stirrer	160 mL	28.8 g[b], [1.5]	10.0 mL[c], [2]	66.7 mL[e], [10]	83.3 mL	
4		10000 mL	1800 g[b], [1.5]	206 mL[d], [2]	417 mL[e], [10]	563 mL	

[a] c_{Al} = 1.44 mol/L; [b] $Al_2(SO_4)_3 \cdot 18\ H_2O$; [c] diluted acetic acid, c = 5.76 mol/L; [d] glacial acetic acid, c = 17.5 mol/L; [e] c_{NaOH} = 12.0 mol/L

2.2.5. $Al_3O(HO_2CCH_3)(O_2CCH_3)_7$ (2)

The optimized synthesis conditions obtained using the HT method (Section S2.1.2) were used for the upscaling in 10 mL Pyrex glass vials and a 250 mL screw-top jar (Table S2.14).

Synthesis of 2 in a 10 mL Pyrex glass vial (Table S2.14-2)
In a 10 mL Duran® glass vial, 262.5 mg (1.26 mmol) aluminum isopropoxide, 4.5 mL (78.8 mmol) glacial acetic acid and 1.5 mL (15.9 mmol) acetic anhydride were mixed. The reaction mixture was heated without stirring to 140 °C for 24 h. After cooling to room temperature the product was separated by filtration and not further purified to yield 262.5 mg (1.33 mmol, 97% based on Al^{3+}, calculated using the composition $Al_3O(HO_2CCH_3)(O_2CCH_3)_7$.

Synthesis of 2 in a 250 mL screw-top jar (Table S2.14-3)
In a 250 mL screw-top jar, 5.25 g (25.7 mmol) aluminum isopropoxide, 90 mL (1.58 mol) glacial acetic acid and 30 mL (317 mmol) acetic anhydride were added. After the jar was sealed the reaction mixture was heated to 140 °C for 24 h. After cooling to room temperature the product was separated by filtration and not further purified to yield 3.78 g (6.63 mmol, 77% based on Al^{3+}, calculated using the composition $Al_3O(HO_2CCH_3)(O_2CCH_3)_7$.

Table S2.14. List of reactor types and starting materials used for the synthesis scale-up of $Al_3O(HO_2CCH_3)(O_2CCH_3)_7$ (**2**) by gradually increasing the size of the batch reactor.

No.	Type of reactor	$V_{solution}$	V (mL); m (g), [n]				T; t
			Al(O-*i*Pr)$_3$	Al	CH_3COOH	$(CH_3CO)_2O$	
**1**	Multiclave with 2 mL Teflon inserts	0.8 mL	35 mg, [171 µmol]	/	600 µL[d], [10.5 mmol]	200 µL, [2.1 mmol]	140 °C; 3-24-3 h
**2**	Pyrex glass tubes	6 mL	262.5 mg, [1.28 mmol]	/	4.5 mL[d], [78.8 mmol]	1.5 mL, [15.9 mmol]	140 °C; 24 h
**3**	Screw-top jar	120 mL	5.25 g, [25.7 mmol]	/	90 mL[d], [1.58 mol]	30 mL, [317 mmol]	

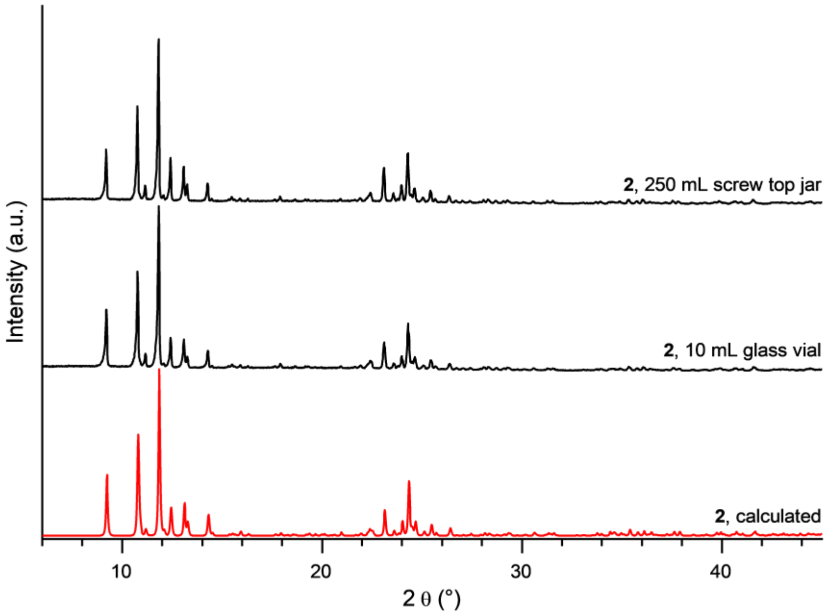

Figure S2.7. Measured and calculated (red line) PXRD patterns of **2** synthesized in10 mL Pyrex glass vials and in a 250 mL screw top jar.

2.2.6. CAU-55-OH (3)

Synthesis optimization for CAU-55-OH (3) in a multiclave with 2 mL Teflon inserts

The synthesis optimization for CAU-55-OH (**3**) was carried out using our custom-made steel autoclaves with 24 Teflon® inserts with a total volume of 2 mL each (Fig. S2.1). Aqueous solutions of $NaAlO_2$ (c = 0.72 mol/L), acetic acid (5.76 mol/L) and NaOH (c = 2 mol/L) were mixed, and the reaction volume was kept constant at 1 mL by adding water. After sealing the autoclaves the reaction vessels were placed in a Memmert UNB 500 oven with forced ventilation and a programmable temperature-time-program. The reaction products were separated by centrifugation (*micro centrifuge Gusto, Heathrow Scientific*) and washed with water and methanol. The optimized synthesis conditions and molar ratios of the starting materials used for the synthesis scale up are given in Table S2.15-1.

Synthesis of 3 in a 10 mL Pyrex glass vial (Table S2.15-2)

In a 10 mL Duran® glass vial, 5 mL of water and 2 mL of an aqueous solution of $NaAlO_2$ (c_{Al} = 0.72 mol/L, 1.44 mmol), were mixed with 1 mL (5.76 mol/L, 5.76 mmol) of diluted acetic acid. Subsequently the reaction vial was sealed and the reaction mixture was heated at 100 °C for 2 h under stirring using a heating plate with magnetic stirrer (MR Hei-Tec, Heidolph). After cooling to room temperature, the reaction product was separated by centrifugation (*Allegra 64R, Beckman Coulter)*, washed with water and methanol and dried at room temperature to yield 24 mg (9.3 µmol, 15% based on Al^{3+}, calculated using the composition $[Al_{24}(OH)_{56}(O_2CCH_3)_{12}](OH)_4 \cdot 14.8\ H_2O$).

Synthesis of 3 in a 250 mL round bottom flask (Table S2.15-3)

In a 250 mL round bottom flask, 2.36 g (28.8 mmol) of $NaAlO_2$ were dissolved in 140 mL of water and 20 mL of diluted acetic acid (5.76 mol/L, 115 mmol) were added. The reaction mixture was heated and stirred under reflux conditions for 2 h using a heating plate with magnetic stirrer (MR Hei-Tec, Heidolph). After cooling to room temperature, the reaction product was separated by centrifugation and washed with water and methanol to yield 276 mg (0.11 mmol, 9% based on Al^{3+}, calculated using the composition $[Al_{24}(OH)_{56}(O_2CCH_3)_{12}](OH)_4 \cdot 14.8\ H_2O$).

Synthesis of 3 in a 10 L round bottom flask (Table S2.15-4)

In a 10 L round bottom flask, 148 g (1.8 mol) of $NaAlO_2$ were dissolved in 9.6 L of water and 413 mL (7.2 mol) of glacial acetic acid were added. The reaction mixture was heated and stirred under reflux conditions for 20 h. After cooling to room temperature, the reaction product was separated by filtration and washed with water and ethanol to yield 16.1 g (154 mmol, 8% based on Al^{3+}, calculated using the composition $[Al_{24}(OH)_{56}(O_2CCH_3)_{12}](OH)_4 \cdot 14.8\ H_2O$).

Table S2.15. List of reactor types and starting materials used for the synthesis scale-up of CAU-55-OH (**3**) by gradually increasing the size of the batch reactor. One equivalent corresponds to a concentration of 1.44 mmol/L.

No.	Type of reactor	$V_{solution}$ (mL)	V (mL); m (g), [equivalents]				T; t
			$NaAlO_2$	CH_3COOH	NaOH	H_2O	
1	multiclave with 2 mL Teflon inserts	1 mL	0.250 mL[a], [1]	0.125 mL[c], [4]	/	0.625 mL	100 °C; 12 h
3	Pyrex glass tubes	8 mL	2.0 mL[a], [1]	1.0 mL[c], [4]		5.0 mL	100 °C; 2 h
4	Round bottom flask with stirrer	160 mL	2.36 g[b], [1]	20.0 mL[c], [4]		140 mL	
5		10000 mL	148 g[b], [1]	413 mL[d], [4]		9600 mL	

[a] c_{Al} = 0.72 mol/L; [b] $NaAlO_2$; [c] diluted acetic acid, c = 5.76 mol/L; [d] glacial acetic acid, c = 17.5 mol/L

3. Crystal structure determination and refinement

3.1. Rietveld, Le Bail plots and crystallographic data

The structural data (Table S3.1 - S3.3) for Al(OH)(O_2CCH_3) (**1A**), CAU-65 (**1B**) and $Al_3O(HO_2CCH_3)(O_2CCH_3)_7$ (**2**) obtained from Rietveld refinements have been deposited with the Cambridge Crystallographic Data Center (CCDC-numbers 2363306, 2363309 and 2363310). CAU-55-OH (**3**) is isostructural to CAU-55-X, previously reported by our group with X = Cl, Br, I, HSO_4[13] and a Le Bail fit was carried out to confirm the phase purity and to determine the lattice parameters (Figure S3.4, Table S3.3).

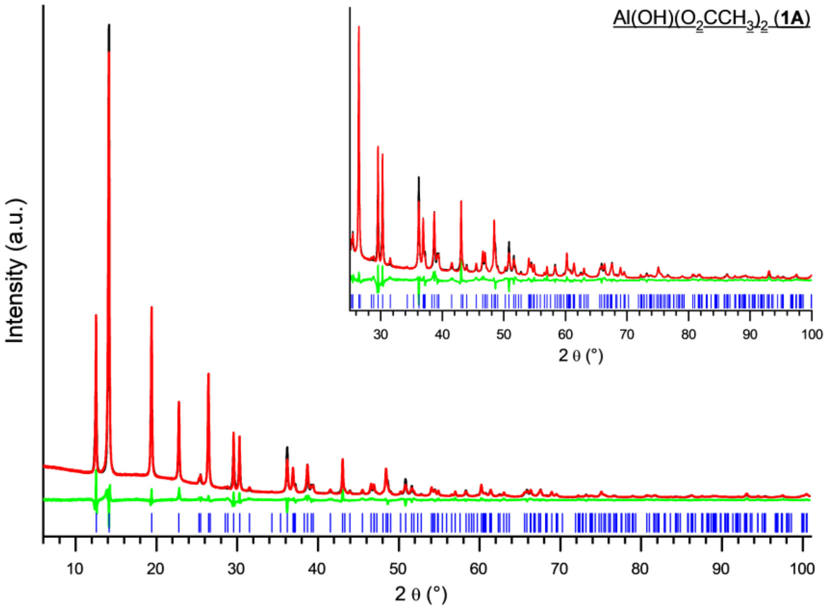

Figure S3.1. Final Rietveld-plot of the structure refinement of Al(OH)(O_2CCH_3) (**1A**). The experimental data (red line), the calculated diffractogram (black line), the resulting difference (green line) and the allowed Bragg reflection positions (blue ticks) are shown.

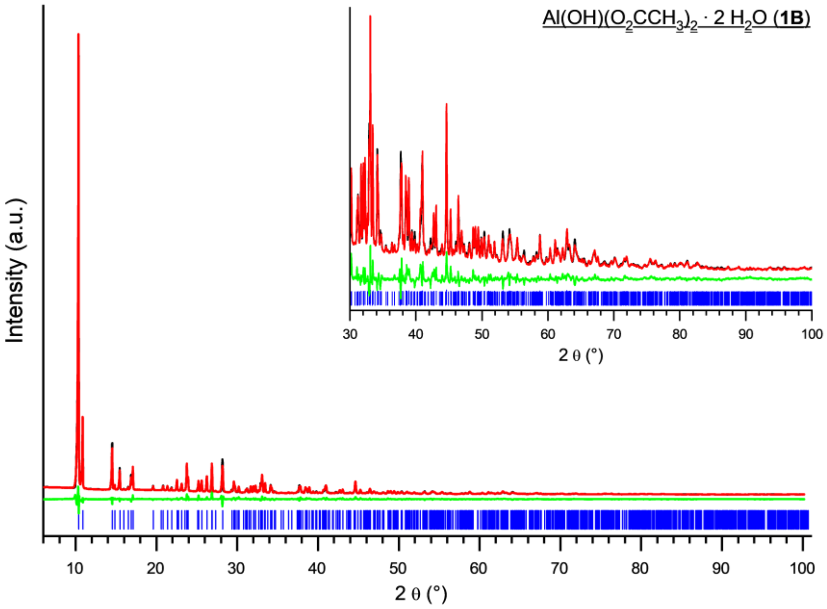

Figure S3.2. Final Rietveld-plot of the structure refinement of CAU-65 (**1B**). The experimental data (red line), the calculated diffractogram (black line), the resulting difference (green line) and the allowed Bragg reflection positions (blue ticks) are shown.

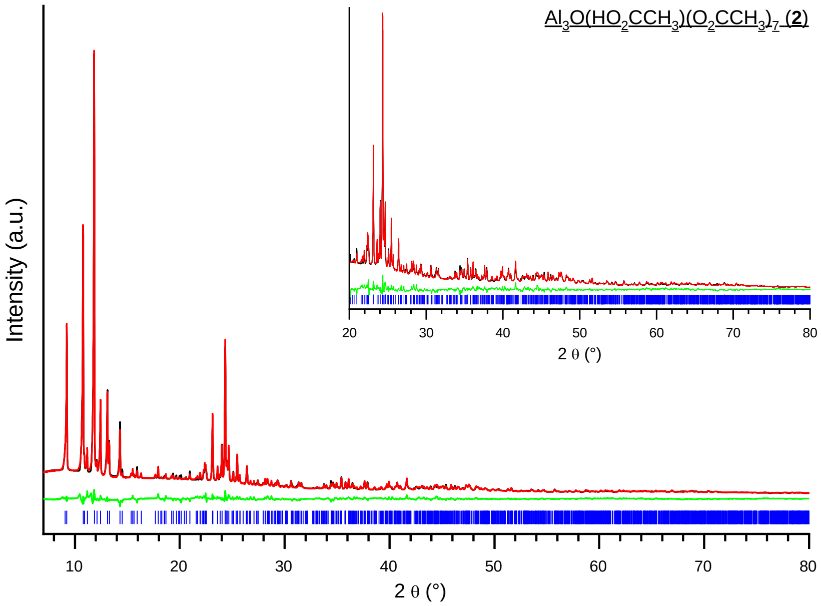

Figure S3.3. Final Rietveld-plot of the structure refinement of $Al_3O(HO_2CCH_3)(O_2CCH_3)_7$ (**2**). The experimental data (red line), the calculated diffractogram (black line), the resulting difference (green line) and the allowed Bragg reflection positions (blue ticks) are shown.

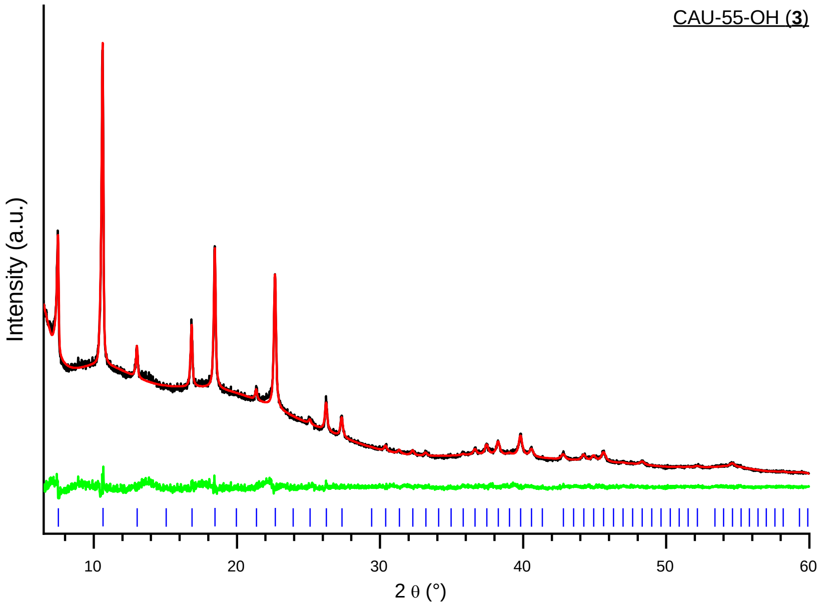

Figure S3.4. Final Le Bail-plot on the PXRD data for CAU-55-OH (**3**). The experimental data (red line), the calculated diffractogram (black line), the resulting difference (green line) and the allowed Bragg reflection positions (blue ticks) are shown.

Table S3.1. Crystallographic data for $Al(OH)(O_2CCH_3)$ (**1A**) and $Al(OH)(O_2CCH_3) \cdot 2\ H_2O$ (**1B**) as determined from 3D ED measurements.

	$Al(OH)(O_2CCH_3)$ (**1A**)	CAU-65 (**1B**)
**Sum formula**	$C_8H_{14}Al_2O_{10}$	$C_{16}Al_4O_{28}$
**Wavelength**	0.0251 Å	0.0251 Å
**Temperature**	98(2) K	293(2) K
**Crystal system**	Orthorhombic	Monoclinic
**Space group**	*Cmcm*	*C*2/*c*
**Unit cell dimensions**	a = 14.714(3) Å<br>b = 7.3020(15) Å<br>c = 6.9380(14) Å<br>$\alpha = \beta = \gamma$ = 90 °	a = 14.258(3) Å<br>b = 12.263(3) Å<br>b = 12.504(3) Å<br>α = 90 °<br>β = 119.06(3) °<br>γ = 90 °
**Volume**	745.4(3) $Å^3$	1911.0(8) $Å^3$
**Z**	2	2
**Theta range for data collection**	0.098 to 0.898°	0.098 to 0.898°
**Index ranges**	$-18 \leq h \leq 18$<br>$-8 \leq k \leq 8$<br>$-8 \leq l \leq 8$	$-17 \leq h \leq 17$<br>$-11 \leq k \leq 10$<br>$-15 \leq l \leq 14$
**Reflections collected**	1351	3107
**Independent reflections**	310<br>[R(int) = 0.2006]	1137<br>[R(int) = 0.1697]
**Completeness (to 0.83 Å resolution)**	72.0 %	58.9 %
**R1 (ED model) [I > 4σ(I)]**	0.3021	0.1908

Table S3.2. Selected crystallographic data and details of the structure refinements for **2** obtained from single crystal X-ray diffraction data. CCDC-2361983 contain the supplementary crystallographic data of **2.**

	$Al_3O(HO_2CCH_3)(O_2CCH_3)_7$ (**2**)
**Empirical formula**	$C_{16}H_{25}Al_3O_{17}$
**Formula weight**	570.30
**Temperature/K**	100.00(10)
**Crystal system**	orthorhombic
**Space group**	Pbca
**a/Å**	15.5174(3)
**b/Å**	15.9288(3)
**c/Å**	19.1786(4)
**α/°**	90
**β/°**	90
**γ/°**	90
**Volume/Å^3**	4740.44(16)
**Z**	8
**ρ_{calc}g/cm^3**	1.598
**μ/mm^{-1}**	2.242
**F(000)**	2368.0
**Crystal size/mm^3**	0.18 × 0.06 × 0.03
**2Θ range for data collection/°**	9.196 to 159.446
**Index ranges**	-19 ≤ h ≤ 19, -20 ≤ k ≤ 18, -24 ≤ l ≤ 22
**Reflections collected**	25330
**Independent reflections**	5079 [R_{int} = 0.0341, R_{sigma} = 0.0159]
**Reflections with [I>=2σ (I)]**	4793
**Data/restraints/parameters**	5079/0/335
**Goodness-of-fit on F^2**	1.058
**Final R indexes [I>=2σ (I)]**	R_1 = 0.0449, wR_2 = 0.1171
**Final R indexes [all data]**	R_1 = 0.0468, wR_2 = 0.1187
**Largest diff. peak/hole / e Å^{-3}**	0.60/-0.48

Table S3.3. Crystallographic data for $Al(OH)(O_2CCH_3)$ (**1A**), CAU-65 (**1B**), $Al_3O(HO_2CCH_3)(O_2CCH_3)_7$ (**2**) and CAU-55-OH (**3**) obtained from the Rietveld refinements [a] or Le Bail fits [b]. The samples were obtained using the optimized reaction conditions listed in Table S2.12 - Table S2.15.

	$Al(OH)(O_2CCH_3)$ (**1A**)[a]	CAU-65 (**1B**)[a]	$Al_3O(HO_2CCH_3)(O_2CCH_3)_7$ (**2**)[a]	CAU-55-OH (**3**)[b]
**Sum formula**	$[Al(OH)(O_2CCH_3)_2]$	$[Al(OH)(O_2CCH_3)_2] \cdot 2\ H_2O$	$Al_3O(HO_2CCH_3)(O_2CCH_3)_7$	$[Al_{24}(OH)_{56}(O_2CCH_3)_{12}](OH)_4 \cdot 16\ H_2O$
**Wavelength** (Å)	1.5406 Å			
**Crystal system**	orthorhombic	monoclinic	orthorhombic	cubic
**Space group**	*Cmcm*	*C*2/*c*	*Pbca*	$Im\bar{3}m$
**Unit cell dimensions** (Å), (°)	*a* = 14.0865(4) Å, *b* = 6.9846(2) Å, *c* = 6.68361(14) Å, *α* = *β* = *γ* = 90 °	*a* = 13.9673(3) Å, *b* = 11.91431(19) Å, *c* = 12.0413(3) Å, *α* = 90 °, *β* = 119.3995(15) °, *γ* = 90 °	*a* = 15.8071(3) Å, *b* = 16.2001(3) Å, *c* = 19.1361(3) Å, *α* = *β* = *γ* = 90 °	*a* = *b* = *c* = 16.6209(8) Å, *α* = *β* = *γ* = 90 °
**Volume** ($Å^3$)	657.59(3)	1745.76(6)	4900.29(14)	4591.64
R_{wp} (%)	7.27	6.40	4.95	2.62
**GoF** (%)	1.33	2.05	0.83	3.17

[a] crystallographic data obtained from Rietveld refinement; [b] crystallographic data obtained from Le Bail fit

3.2. Crystal structure of Al(OH)(O_2CCH_3)$_2$ (1A)

The compound Al(OH)(O_2CCH_3)$_2$ (**1A**) crystallizes in the orthorhombic crystal system and the Al^{3+} ions in the crystal structure of Al(OH)(O_2CCH_3)$_2$ (**1A**) are octahedrally surrounded by six oxygen atoms with four oxygen atoms from carboxylate groups of four different acetate ions and two oxygen atoms from μ_2-OH groups (Figure S3.5 (a)). The asymmetric unit consists of one Al^{3+} ion, one coordinating acetate ion as well as one coordinating μ_2-OH group (Figure S3.5 (b)). The [AlO_6] octahedrons are *trans*-corner-linked through μ_2-OH groups forming linear chains of corner-sharing octahedrons along the crystallographic *c*-axis (Figure S3.5 (c)). The chains are alternately staggered along the *a*- and *b*-axis, resulting in a dense packing of the chains in the crystal structure of **1A** (Figure S3.5 (d) and (e)).

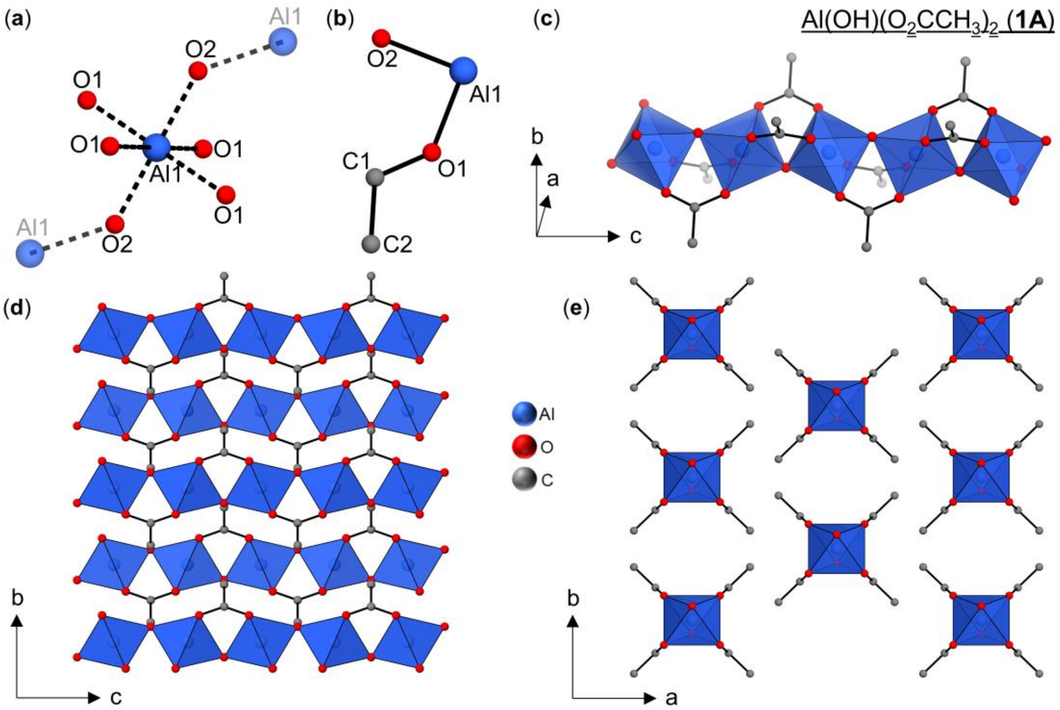

Figure S3.5. Crystal structure of Al(OH)(O_2CCH_3)$_2$ (**1A**). (**a**) Octahedral coordination environment of Al^{3+} ions, (**b**) asymmetric unit of the structure, (**c**) 1D linear rod-shaped building unit formed by *trans*-corner-sharing [AlO_6]-octahedra connected through μ_2-OH-groups. (**d**) and (**e**): Crystal structure of **1A** as seen along the *a*-axis (**d**) and the *c*-axis (**e**).

Crystal structure of CAU-65 (1B)

The compound CAU-65 (**1B**) crystallizes in the monoclinic crystal system and the Al^{3+} ions in the crystal structure of $Al(OH)(O_2CCH_3)_2 \cdot 2\ H_2O$ (**1B**) are octahedrally surrounded by six oxygen atoms with four oxygen atoms from carboxylate groups of four different acetate ions and two oxygen atoms from μ_2-OH groups (Figure S3.6 (a)). The asymmetric unit consists of two Al^{3+} ions, two coordinating acetate ions as well as one coordinating μ_2-OH group (Figure S2.4.1.(b)). Additionally, there are two water molecules located in the pores of **1B**. The $[AlO_6]$ octahedrons alternately *cis*- and *trans*-corner linked through μ_2-OH groups forming infinite zigzag chains of corner-sharing octahedrons along the crystallographic *c*-axis (Figure S3.6 (e)). The chains are alternately staggered along the *a*- and *b*-axis, resulting in pore channels between the chains occupied by two water molecules per formula unit (Figure S3.6 (f) and (g)). The water molecules form characteristic hydrogen bonds with neighboring water molecules, coordinated μ_2-OH groups and coordinating carboxylate groups (Figure S3.6 (c) and (d)).

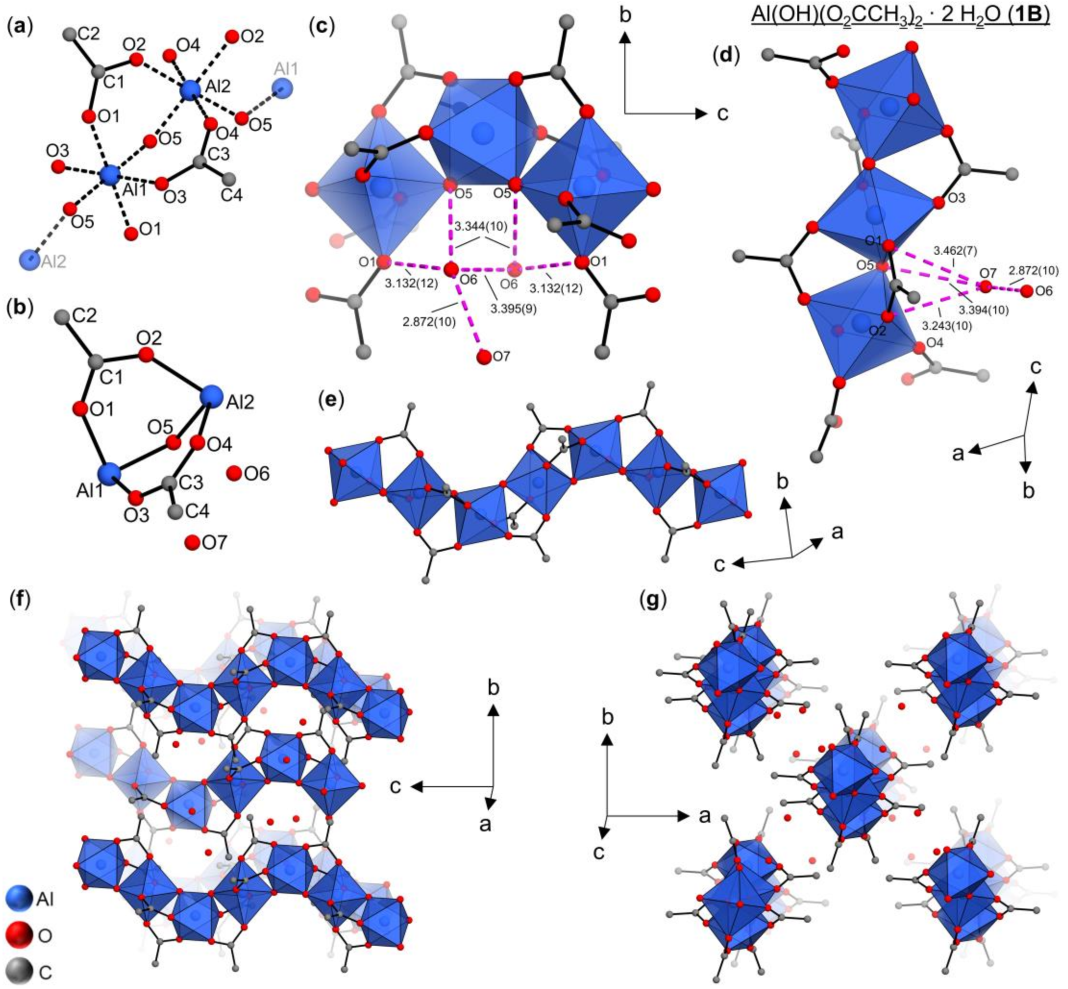

Figure S3.6. Crystal structure of CAU-65 (**1B**). (**a**) Octahedral coordination environments of Al^{3+} ions, (**b**) asymmetric unit; (**c**) and (**d**) Characteristic hydrogen bonds found in CAU-65 (**1B**). The hydrogen donor-acceptor distances were calculated using structural data, determined from Rietveld-refinement against X-ray diffraction data. (**e**) 1D zigzag-chains formed by alternating *cis*- and *trans*-corner-sharing $[AlO_6]$-octahedra connected through μ_2-OH-groups. (**f**) and (**g**): Crystal structure of **1B** as seen along the *a*-axis (**d**) and the *c*-axis (**e**).

3.3. Crystal structure of $Al_3(O)(HO_2CCH_3)(O_2CCH_3)_7$ (2)

The compound $Al_3(O)(HO_2CCH_3)(O_2CCH_3)_7$ (**2**) crystallizes in the orthorhombic crystal system and is composed of trimeric μ_3-O centered building units (Figure S3.7d). These trinuclear units consist of three corner-sharing $[AlO_6]$ octahedral which are connected by six acetate groups, leading to the composition $(Al_3(\mu_3\text{-}O)(OOCCH_3))^+$. An additional end-on-binding protonated acetate ligand forms hydrogen bonds with adjacent oxygen atoms of the acetate groups. The trinuclear units are bridged by two terminal acetate ions which leads to the formation of a zig-zag chain structure (Figure S3.7c).

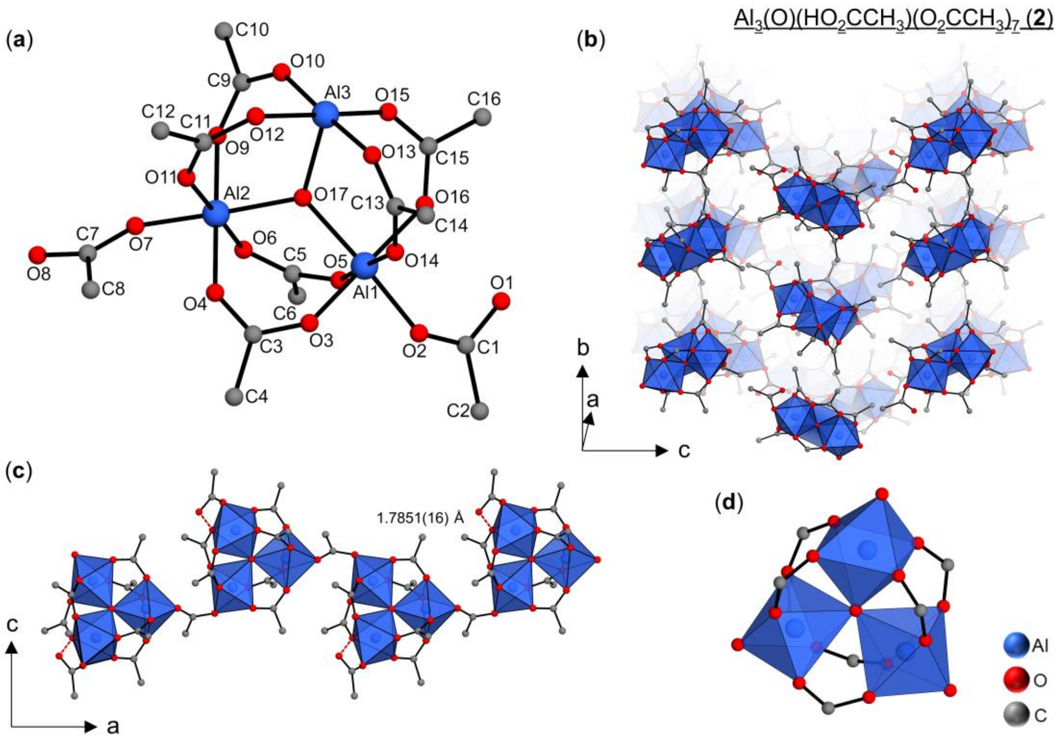

Figure S3.7. Crystal structure of $Al_3(O)(HO_2CCH_3)(O_2CCH_3)_7$ (**2**). (**a**) Asymmetric unit, (**d**) trinuclear μ_3-O-centered building unit composed of three corner-sharing $[AlO_6]$-octahedra connected through six acetate groups. (**b**) Crystal structure of **2** as seen along the *a*-axis. (**d**) Chains of trinuclear building units along the *a*-axis connected through acetate ions.

3.4. Crystal structure of CAU-55-OH $[Al_{24}(OH)_{56}(O_2CCH_3)_{12}](OH)_4$ (3)

Based on the PXRD (Figure S3.8) data and the composition we anticipate that compound $[Al_{24}(OH)_{56}(O_2CCH_3)_{12}](OH)_4)$ (**3**) is isostructural to CAU-55-X[13] previously described by our group. In the following section, the crystal structure of CAU-55-X with X^- = OH^-, HSO_4^-, Cl^-, Br^- and I^- is described. CAU-55-X crystallizes in the cubic crystal system and the Al^{3+} ions are octahedrally surrounded by six oxygen atoms with one oxygen atom from carboxylate groups of an acetate ion, four oxygen atoms μ_2-OH groups and one oxygen atom from an μ_3-OH groups. The asymmetric unit consists of one Al^{3+} ion, one coordinating acetate ion, two coordinating μ_2-OH groups as well as one coordinating μ_3-OH group (Figure S3.9 (a)). Additionally, there are three water molecules and one X^- ion (OH^-) located in the pores. The compound contains $[Al_{24}(OH)_{56}(CH_3COO)_{12}]^{4+}$ ions composed of $\{Al_3(\mu_3\text{-OH})(\mu_2\text{-OH})_3\}^{5+}$ trinuclear building units. In the Al_{24} ions, edge sharing of adjacent trinuclear units through µ-OH-groups is observed leading to a cube consisting of eight trinuclear clusters occupying the corners of the cube (Figure S3.9 (c)). Thus, the structure of the Al_{24} ion is related to a cuboctahedron, with the 8 triangular faces occupied by the trinuclear clusters, the 6 square faces parallel to the pore windows and the 12 vertices occupied by the acetate ions. In the crystal structure of CAU-55-X, a body-centered cubic packing of the Al_{24} ions is found. The counter ions occupy the octahedral voids and due to favorable interactions with the μ-OH sites on the cage windows, a shift of X^- ions from the ideal position (½ 0 ½ and ½ 0 0) to (½ y ½ and x 0 0) towards the pore windows is found (Figure S3.9 (e)). For charge balance 2/3 of this position is occupied by counter ions (X^-) is found in CAU-55-X and for 1/3 of the positions the counter ions are replaced by water molecules.

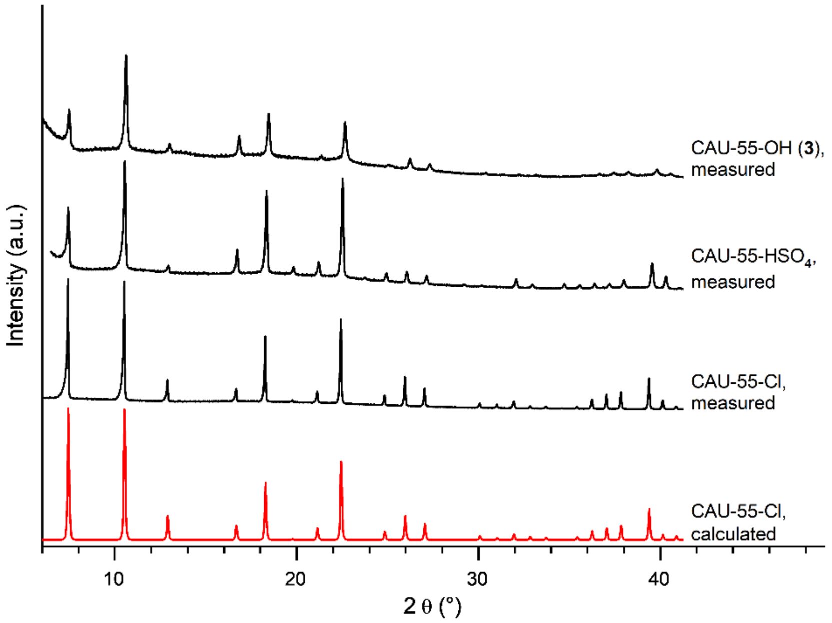

Figure S3.8. Measured and calculated (red line) PXRD patterns of CAU-55-X[13], with X = Cl^-, HSO_4^- and OH^- (**3**).

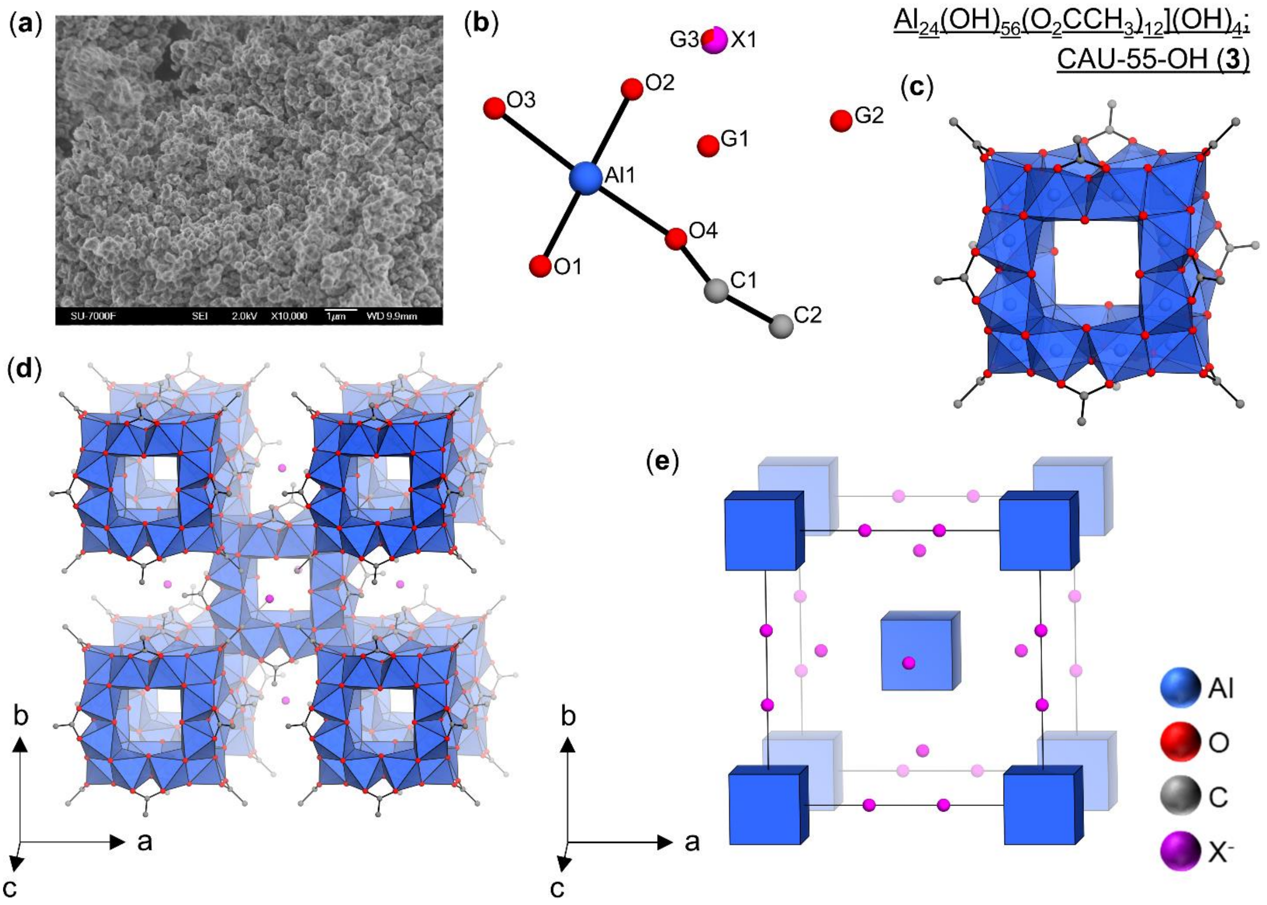

Figure S3.9. (**a**) Scanning electron micrographs of **3** acquired at 3000 times magnification. (**b**) - (**c**) Crystal structure of CAU-55-X ($[Al_{24}(OH)_{56}(O_2CCH_3)_{12}](X)_4$). (**b**) Asymmetric unit. (**c**) Al_{24} cluster ion $\{[Al_{24}(OH)_{56}(O_2CCH_3)_{12}]^{4+}\}$ composed of eight edge-sharing trinuclear building units occupying the corners of a cube. (**d**) and (**e**): Body centered packing of Al_{24} cluster cations and X^- (OH^-)-ions occupying the octahedral voids

4. Spectroscopic characterization, elemental analyses and thermal properties

4.1. IR spectroscopy

IR-spectra of the compounds (Figure S4.1) were collected using a Bruker ALPHA-FT-IR A220/D-01 with an ATR-unit. The characteristic vibration bands are assigned in Table S4.1.[14,15]

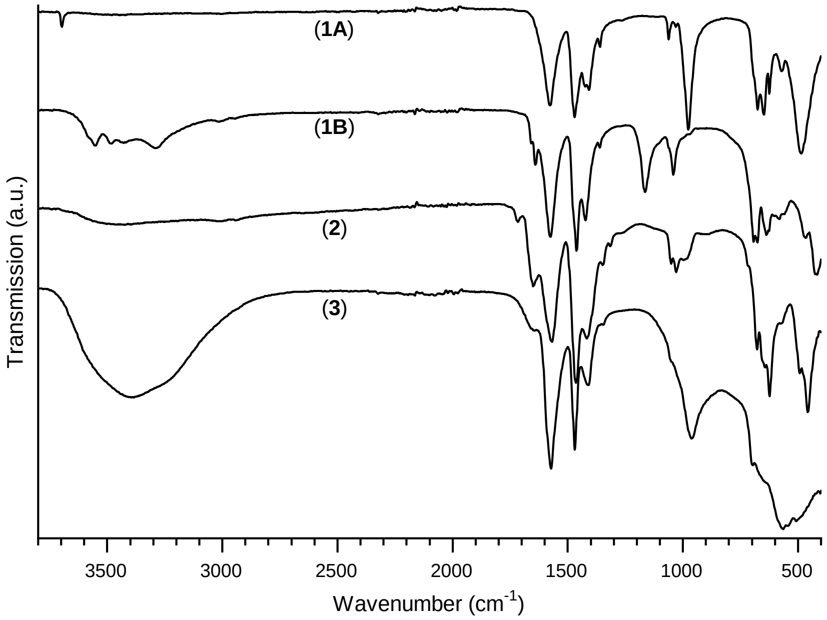

Figure S4.1. FT-IR spectra of the four title compounds (**1A**, **1B**, **2** and **3**). The characteristic vibration bands are assigned in Table S4.1.

Table S4.1. Assignment of the vibrational bands of the title compounds identified in the IR spectra (Figure S4.1).

**Vibration** (cm^{-1})	$Al(OH)(O_2CCH_3)$ (**1A**)	CAU-65 (**1B**)	$Al_3O(HO_2CCH_3)(O_2CCH_3)_7$ (**2**)	CAU-55-OH (**3**)
ν_{as}(OH) [H_2O]	/	3100-3600	/	3000-3700
ν_{as}(OH) [μ-OH]	3698	/	/	/
ν_{as}(C-H) [CH_2; CH_3]	3001	3001	3003	/
ν_s(C-H) [CH_2; CH_3]	/	2944	2940	/
ν_{as}(C=O) [CO_2H]	/	/	1713	/
ν_s(C=O) [CO_2H]	/	1639	1652	/
ν_{as}(C=O) [CO_2^-]	1578	1576	1568	1574
ν_s(C=O) [CO_2^-]	1470	1462	1464	1467
δ_{as}(C-H) [CH_3]	1408	1423	1417	1410
δ(C-H) [CH_3]	673	692	679	698

4.2. Elemental and EDX analyzes

Elemental analyzes and EDX analyzes were carried out to determine the compositions of the title compounds (Table S4.2). The results were compared to the theoretical values calculated using the proposed sum formulas (Table S4.2). Small differences in the measured and calculated carbon and hydrogen content for **2** can be attributed to residues of acetic acid in the sample.

Table S4.2. Results of elemental analyzes and EDX analyzes of (**1A**), (**1B**), (**2**) and (**3**). The theoretical values assuming the proposed sum formulas in Table S4.3 are given in square brackets.

	$Al(OH)(O_2CCH_3)$ (**1A**)	CAU-65 (**1B**)	$Al_3O(HO_2CCH_3)(O_2CCH_3)_7$ (**2**)	CAU-55-OH (**3**)
**Al**[a] (%)	100 [100]	100 [100]	100 [100]	98.5 ± 2.4 [100]
**Na**[a] (%)	/	/	/	1.5 ± 1.7 [0.0]
**S**[a] (%)	/	/	/	/
**C**[b] (%)	28.5 [28.4]	22.8 [22.6]	27.36 [33.69]	11.6 [10.8]
**H**[b] (%)	4.2 [4.6]	5.5 [5.9]	5.14 [4.42]	5.6 [4.8]

[a] element content determined by EDX analyses; [b] element content determined by elemental analyses

4.3. Thermogravimetric curves

Thermogravimetric measurements (Figure S4.2 – S4.5) were performed to determine the solvent content, the thermal stability and the composition of the samples. The thermogravimetric measurements were performed on a Linseis STA PT 1000 (airflow = 6 dm^3/h, heating rate = 4 K/min or 8 K/min). The sample amount was approximately 25 mg for each sample. PXRD patterns collected after the measurements indicated the formation of a reaction product of low crystallinity, which can be assigned to Al_2O_3 for all compounds.

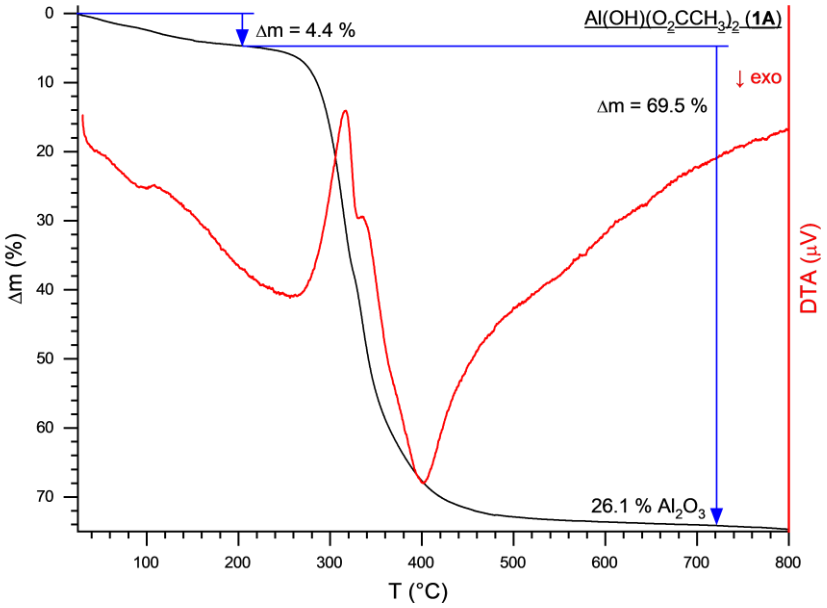

Figure S4.2. Thermogravimetric curve (black), DTA curve (red) and decomposition steps (blue) of $Al(OH)(O_2CCH_3)$ (**1A**) measured with a heating rate of 8 K/min in air atmosphere.

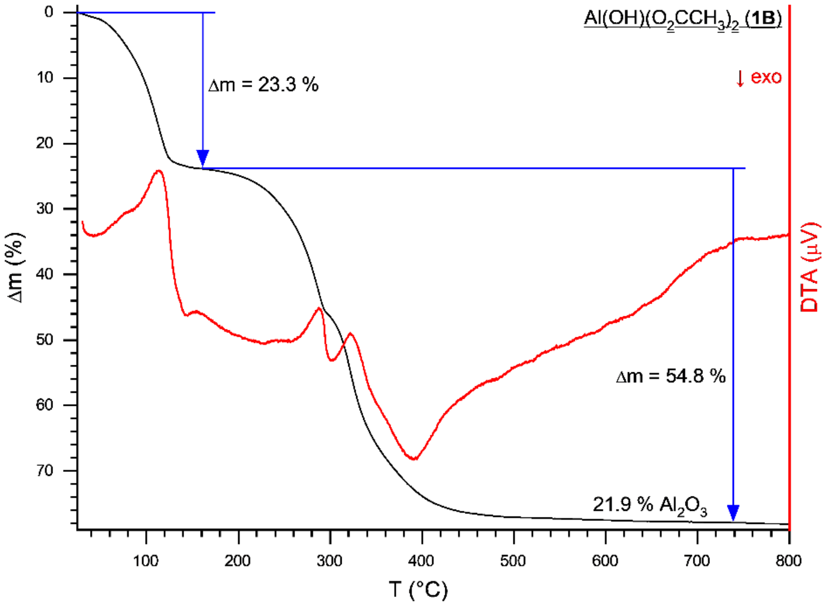

Figure S4.3. Thermogravimetric curve (black), DTA curve (red) and decomposition steps (blue) of CAU-65 (**1B**) measured with a heating rate of 4 K/min in air atmosphere.

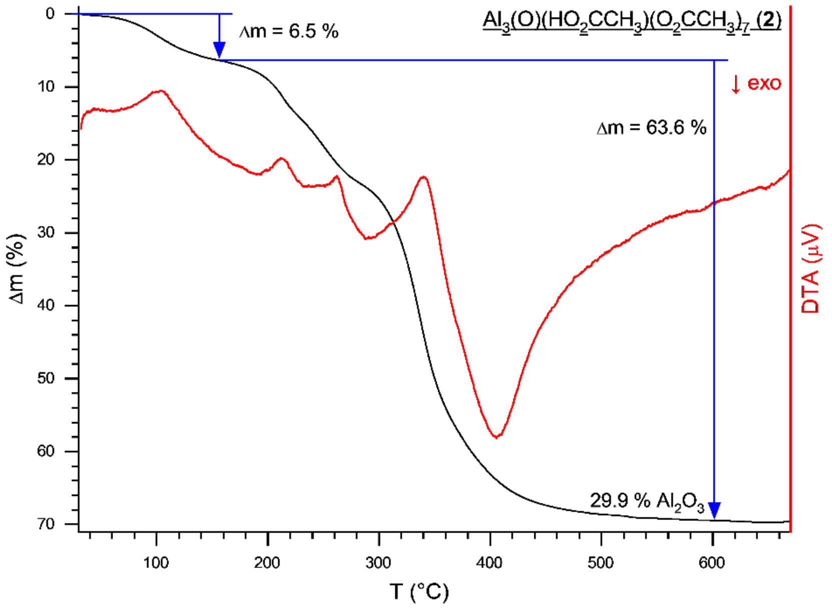

Figure S4.4. Thermogravimetric curve (black), DTA curve (red) and decomposition steps (blue) of $Al_3O(HO_2CCH_3)(O_2CCH_3)_7$ (**2**) measured with a heating rate of 8 K/min in air atmosphere.

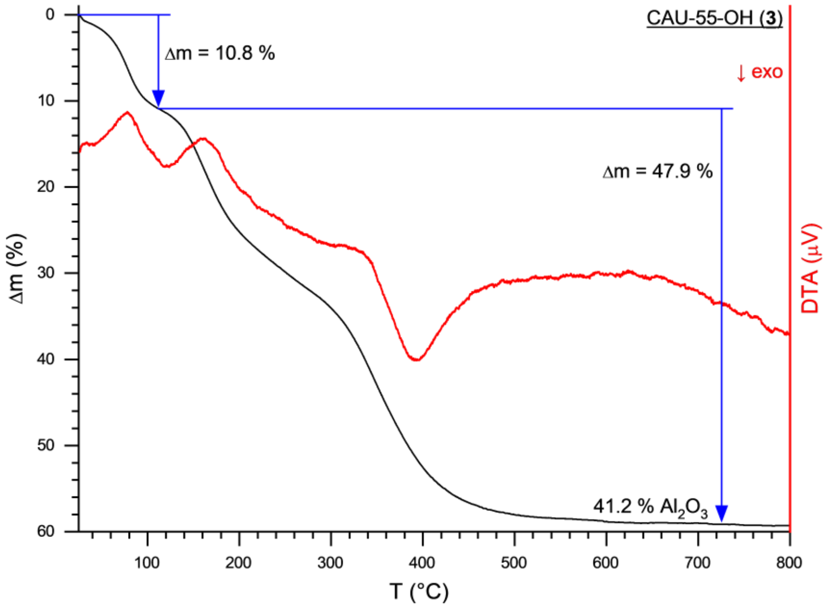

Figure S4.5. Thermogravimetric curve (black), DTA curve (red) and decomposition steps (blue) of CAU-55-OH (**3**) measured with a heating rate of 4 K/min in air atmosphere.

Table S4.3. Decomposition steps of the title compounds, determined by thermogravimetric measurements (Figure S4.2 – S4.5). The theoretical values assuming the sum formulas in Table S4.4 are given in square brackets.

	$Al(OH)(O_2CCH_3)$ (**1A**)	CAU-65 (**1B**)	$Al_3O(HO_2CCH_3)(O_2CCH_3)_7$ (**2**)	CAU-55-OH (**3**)
**$\Delta m_{solvent}$** (%)	4.4 [4.3]	23.8 [23.7]	6.5 [0.0]	10.8 [10.8]
**$\Delta m_{framework}$** (%)	69.5 [65.6]	54.2 [52.3]	63.6 [73.2]	47.9 [43.5]
**$m_{residue}$** (%)	26.1 [30.1]	22.0 [23.9]	29.9 [26.8]	41.2 [45.9]

4.4. Variable temperature PXRD (VT-PXRD)

The Variable temperature powder X-ray diffraction data (VT-PXRD) for **1B** and **2** are shown and discussed in the main manuscript (Figure 5). Additionally, VT-PXRD-data of **1A** and **3** shown in Figure S4.6 and Figure S4.8.

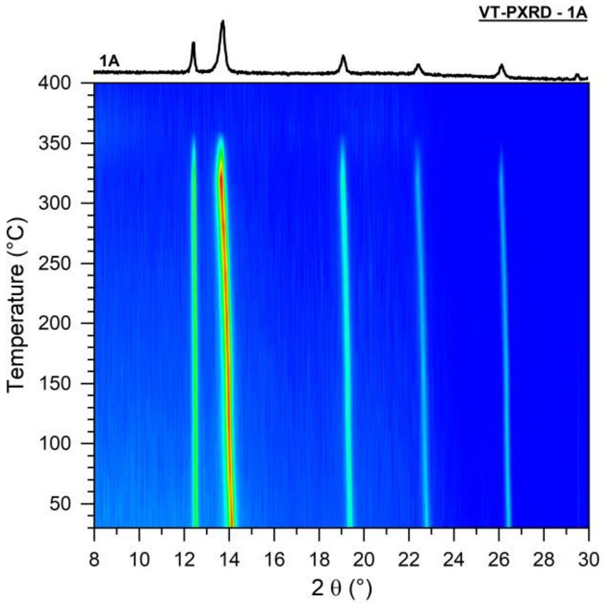

Figure S4.6. Variable temperature powder X-ray diffraction data of Al(OH)(O_2CCH_3) (**1A**). The sample was placed in an open 0.5 mm quartz capillary and heated in 40°C steps up to 400 °C. Additionally, the PXRD data of the as-synthesized product of **1A** is shown.

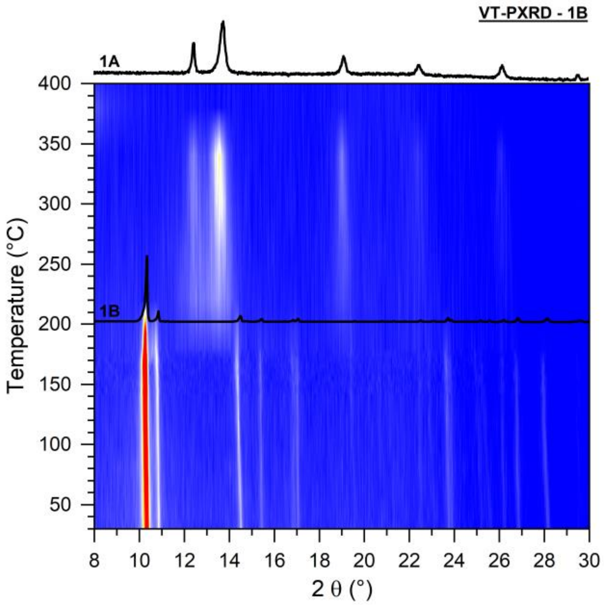

Figure S4.7. Variable temperature powder X-ray diffraction data of CAU-65 (**1B**). The sample was placed in an open 0.5 mm quartz capillary and heated in 20°C steps up to 400 °C. Additionally, the PXRD data of the as-synthesized product of **1B** is shown.

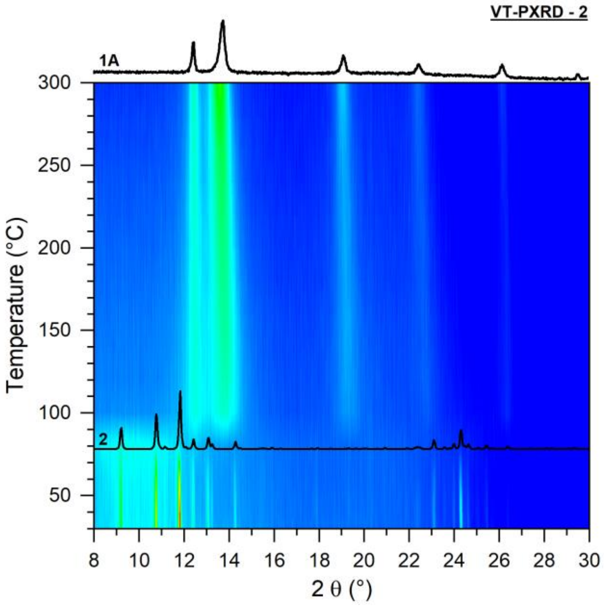

Figure S4.8. Variable temperature powder X-ray diffraction data of $Al_3O(HO_2CCH_3)(O_2CCH_3)_7$ (**2**). The sample was placed in an open 0.5 mm quartz capillary and heated in 20°C steps up to 300 °C. Additionally, the PXRD data of the as-synthesized product of **2** is shown.

4.5. Compositions of the compounds

The compositions of the title compounds (Table S4.4.) were derived under consideration of the crystal structures, thermogravimetric measurements (Figure S4.2 – S4.5), elemental analysis and EDX measurements (Table S4.2).

Table S4.4. Derived compositions of the title compounds, determined under consideration of the SCXRD, thermogravimetric measurements, elemental analysis and EDX measurements.

compound	proposed sum formula	**M** (g/mol)
$Al(OH)(O_2CCH_3)$ (**1A**)	$[Al(OH)(O_2CCH_3)] \cdot 0.4\ H_2O$	169.3
CAU-65 (**1B**)	$[Al(OH)(O_2CCH_3)] \cdot 2.8\ H_2O$	212.5
$Al_3O(HO_2CCH_3)(O_2CCH_3)_7$ (**2**)	$[Al_3O(HO_2CCH_3)(O_2CCH_3)_7]$	570.3
CAU-55-OH (**3**)	$[Al_{24}(OH)_{56}(O_2CCH_3)_{12}](OH)_4 \cdot 16\ H_2O$	2664.8

5. Sorption properties

Nitrogen and vapor (H_2O, CH_3OH, C_2H_5OH, $C_6H_5CH_3$) sorption measurements were carried out using a BEL Japan Inc. BELSORP-max with nitrogen gas at 77 K and water vapor at 298 K. Prior to the measurements the samples were treated for 4 h at elevated temperatures under reduced pressure ($p < 10^{-2}$ mbar).
To confirm the integrity and the long-range order of the structures after thermal activation, PXRD patterns of the samples were collected after sorption measurements (Figure S5.5 – S5.7).

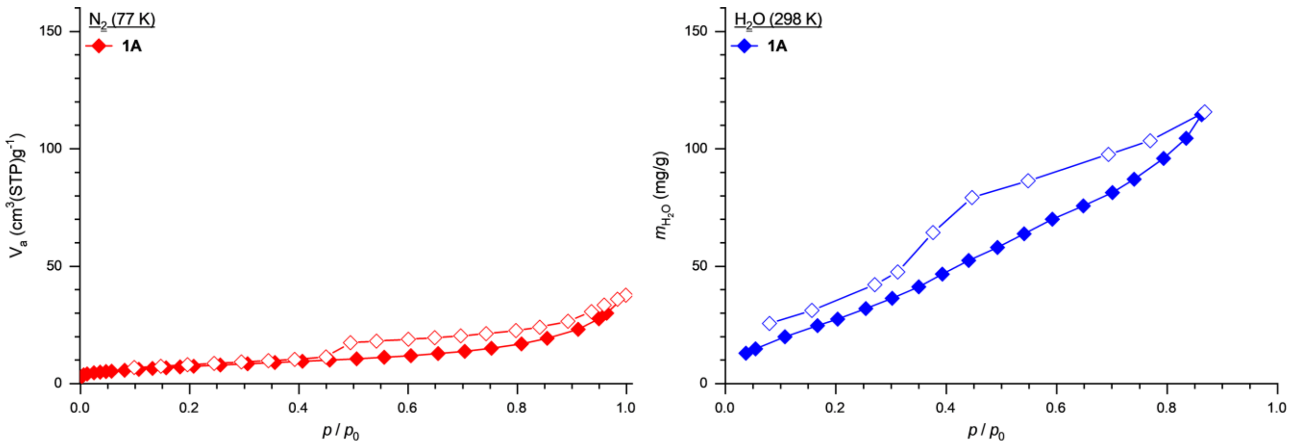

Figure S5.1. N_2 sorption (left) and H_2O sorption (right) isotherm of Al(OH)(O_2CCH_3) (**1A**) collected at 77 K and 298 K, respectively. Prior to the measurements the samples were activated at 70 °C under reduced pressure.

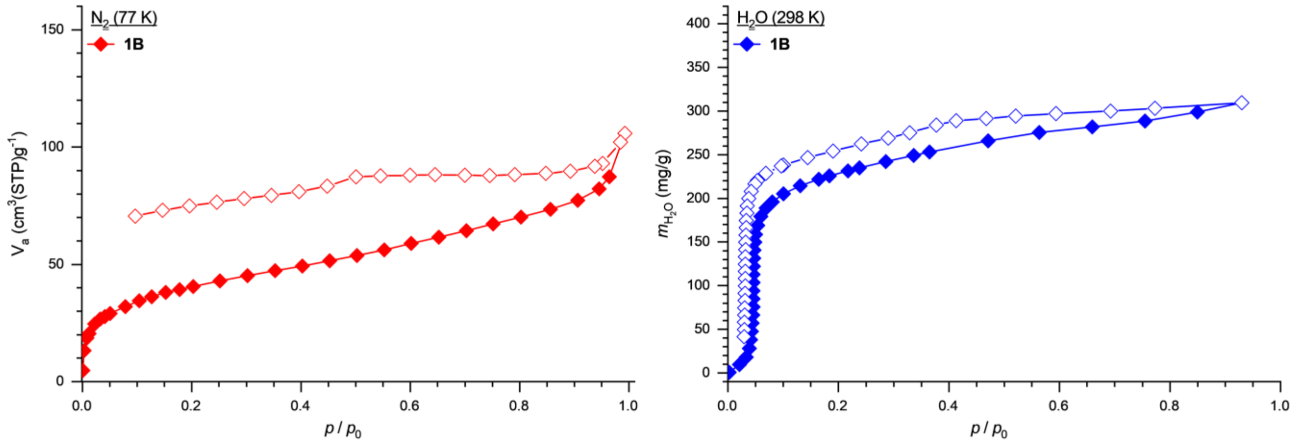

Figure S5.2. N_2 sorption (left) and H_2O sorption (right) isotherm of CAU-65 (**1B**) collected at 77 K and 298 K, respectively. Prior to the measurements the samples were activated at 70 °C under reduced pressure.

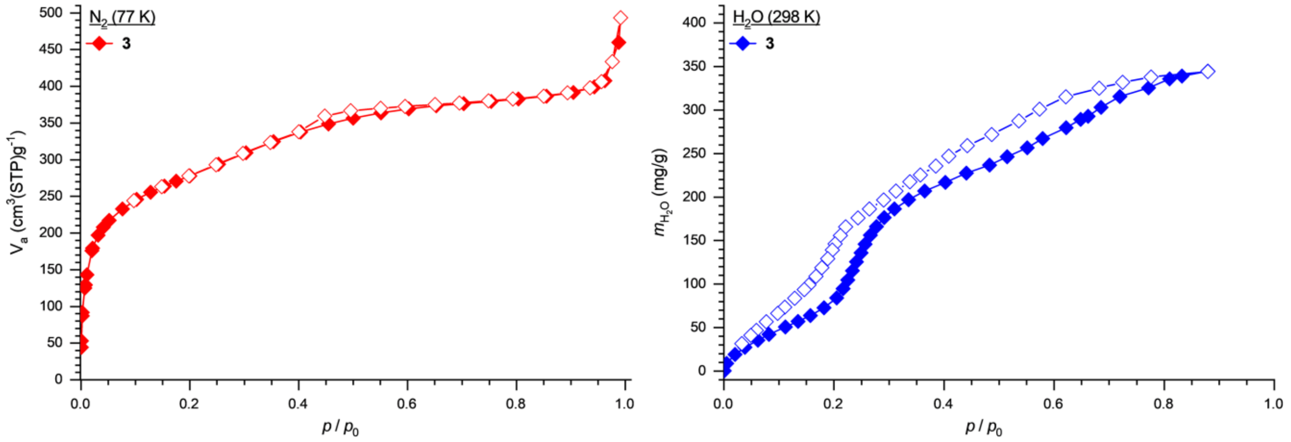

Figure S5.3. N_2 sorption (left) and H_2O sorption (right) isotherm of CAU-55-OH (**3**) collected at 77 K and 298 K, respectively. Prior to the measurements the samples were activated at 70 °C under reduced pressure.

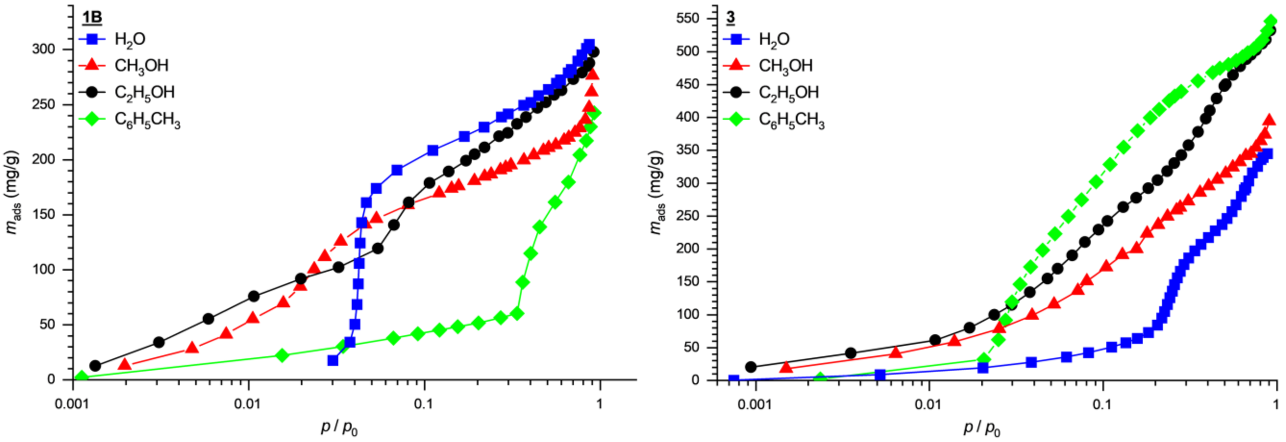

Figure S5.4. Vapor sorption isotherms of CAU-65 (**1B**) (left) and CAU-55-OH (**3**) (right) plotted with a logarithmic x-axis collected at 298 K, respectively. Prior to the measurements the samples were activated at 70 °C under reduced pressure.

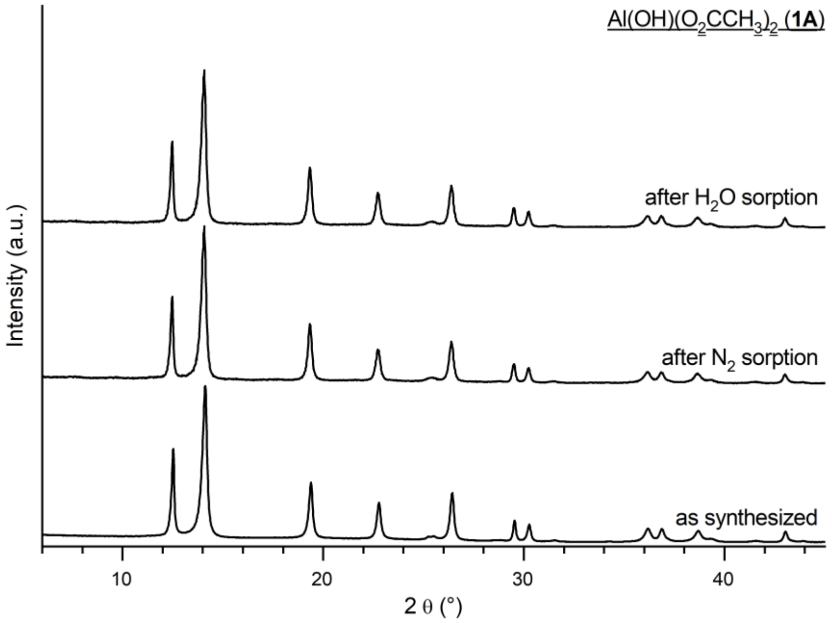

Figure S5.5. PXRD pattern of Al(OH)(O_2CCH_3) (**1A**) after N_2 and H_2O sorption experiments collected at 77 K and 298 K. The samples were activated at 70 °C for 4 h under reduced pressure prior the measurements.

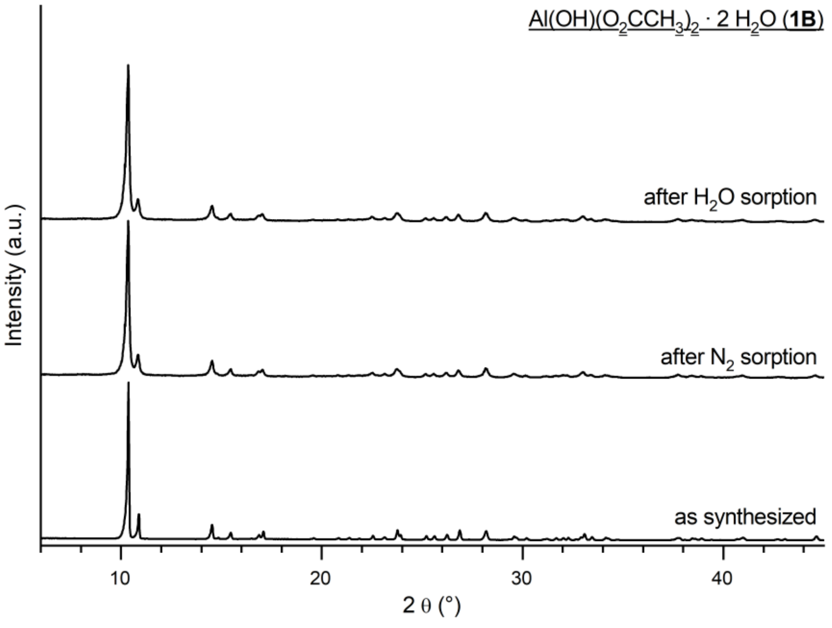

Figure S5.6. PXRD pattern of CAU-65 (**1B**) after N_2 and H_2O sorption experiments collected at 77 K and 298 K. The samples were activated at 70 °C for 4 h under reduced pressure prior the measurements.

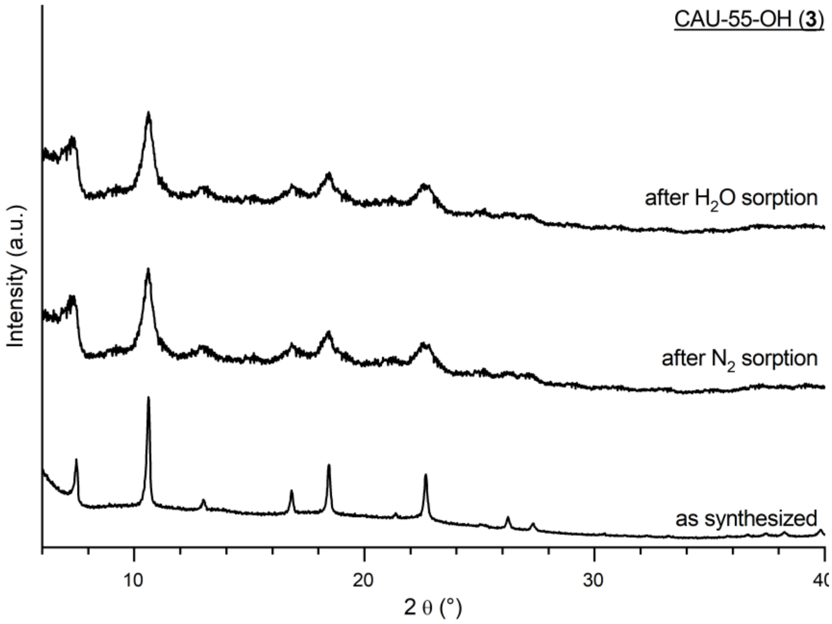

Figure S5.7. PXRD pattern of CAU-55-OH (**3**) after N_2 and H_2O sorption experiments collected at 77 K and 298 K. The samples were activated at 70 °C for 4 h under reduced pressure prior the measurements.

5.1. Crystallographic data for 1B containing guest molecules

In addition to the sorption experiments, powder X-ray diffraction data of **1B** after the adsorption of different organic vapors were collected (Figure 5e). The samples were placed in an open 0.7 mm glass capillary and activated at 70 °C under reduced pressure. Subsequently, 50 µL of methanol (CH_3OH), ethanol (C_2H_5OH) or toluene ($C_6H_5CH_3$) were centrifuged into the capillary.
The structural data for CAU-65 (**1B**) containing different quest molecules (Table S5.1) obtained from Rietveld refinements (Figure S5.9 - S5.10) have been deposited with the Cambridge Crystallographic Data Center (CCDC-numbers 2363307-2363308). For CAU-65 (**1B**) activated or at 70°C under reduced pressure and CAU-65 (**1B**) containing toluene as the guest molecules a Le Bail fit was carried out to confirm the phase purity and to determine the lattice parameters (Figure S5.8 and S5.11, Table S5.1).

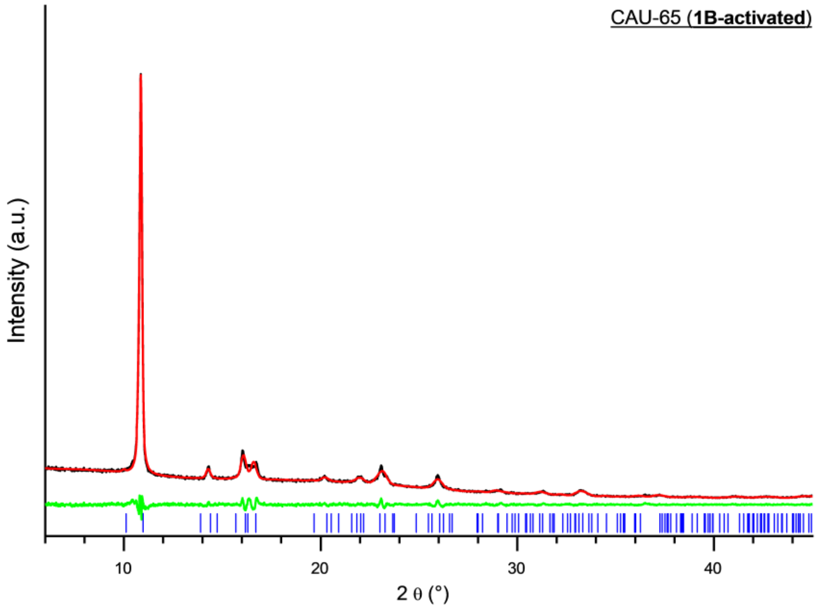

Figure S5.8. Final Le Bail-plot on the PXRD of CAU-65 after the adsorption of toluene (**1B-Toluene**). Prior to the measurement, the sample was placed in an open 0.7 mm glass capillary and activated at 70 °C under reduced pressure. The experimental data (red line), the calculated diffractogram (black line), the resulting difference (green line) and the allowed Bragg reflection positions (blue ticks) are shown.

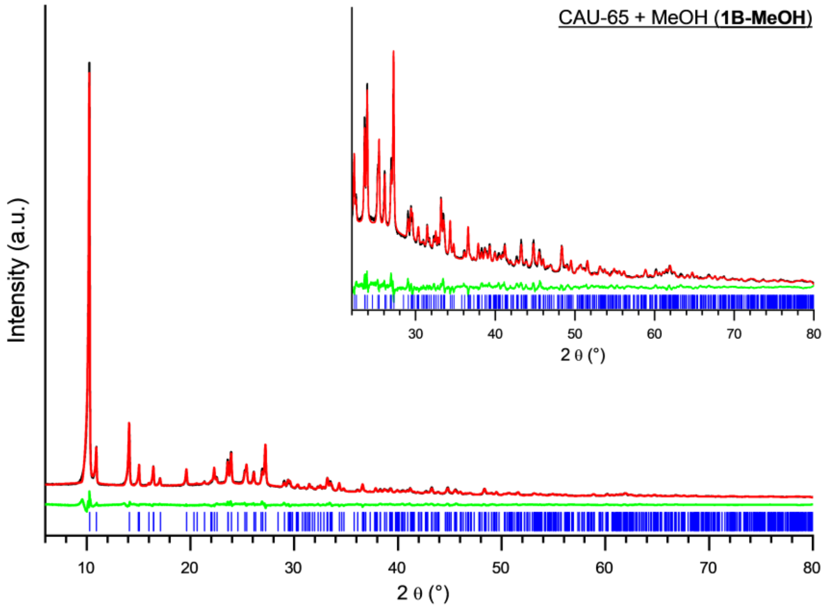

Figure S5.9. Final Rietveld-plot of the structure refinement of CAU-65 after the adsorption of methanol (**1B-MeOH**). Prior to the measurement, the sample was placed in an open 0.7 mm glass capillary and activated at 70 °C under reduced pressure. Subsequently, 50 µL of methanol (CH_3OH) was centrifuged into the capillary. The experimental data (red line), the calculated diffractogram (black line), the resulting difference (green line) and the allowed Bragg reflection positions (blue ticks) are shown.

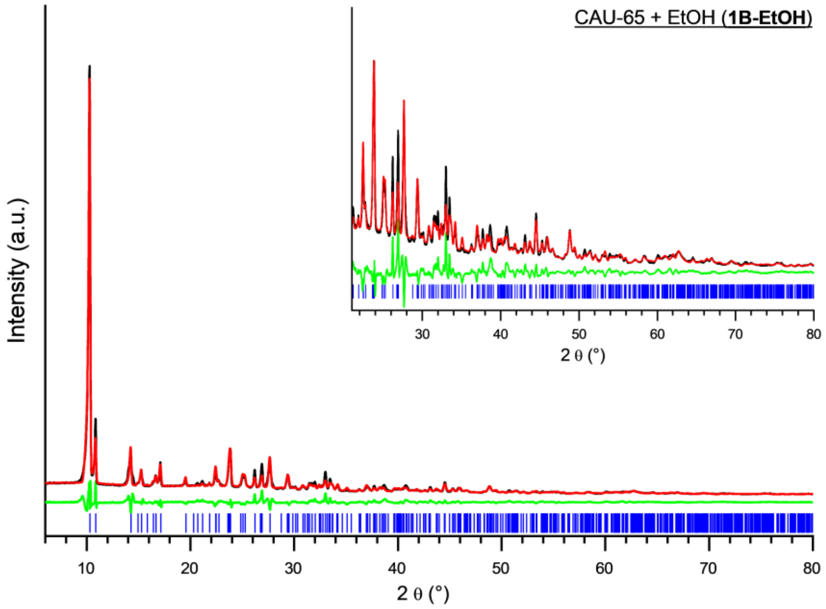

Figure S5.10. Final Rietveld-plot of the structure refinement of CAU-65 after the adsorption of ethanol (**1B-EtOH**). Prior to the measurement, the sample was placed in an open 0.7 mm glass capillary and activated at 70 °C under reduced pressure. Subsequently, 50 µL of ethanol (C_2H_5OH) was centrifuged into the capillary. The experimental data (red line), the calculated diffractogram (black line), the resulting difference (green line) and the allowed Bragg reflection positions (blue ticks) are shown.

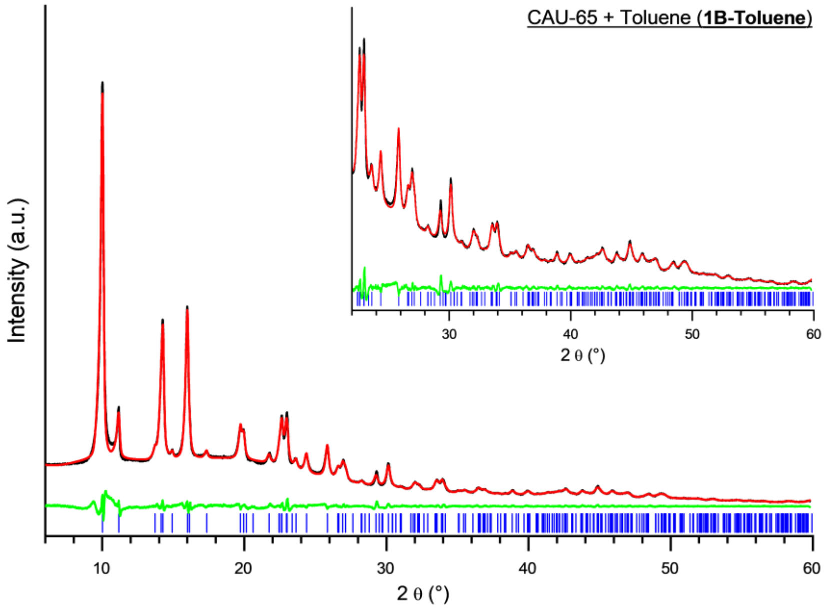

Figure S5.11. Final Le Bail-plot on the PXRD of CAU-65 after the adsorption of toluene (**1B-Toluene**). Prior to the measurement, the sample was placed in an open 0.7 mm glass capillary and activated at 70 °C under reduced pressure. Subsequently, 50 µL of toluene ($C_6H_5CH_3$) was centrifuged into the capillary. The experimental data (red line), the calculated diffractogram (black line), the resulting difference (green line) and the allowed Bragg reflection positions (blue ticks) are shown.

Table S5.1. Crystallographic data for Al(OH)(O_2CCH_3) · x Guest (**1B**) obtained from the Rietveld refinements[a] or Le Bail fits[b].

	Al(OH)(O_2CCH_3) (**1B-activated**)[b]	Al(OH)(O_2CCH_3) · 2 H_2O (**1B**)[a]	Al(OH)(O_2CCH_3) · 1 CH_3OH (**1B-MeOH**)[a]	Al(OH)(O_2CCH_3) · 2 C_2H_5OH (**1B-EtOH**)[a]	Al(OH)(O_2CCH_3) · 2 $C_6H_5CH_3$ (**1B-Toluene**)[b]
**CCDC-number**	/	2363306	2363308	2363307	/
**Sum formula**	[Al(OH)(O_2CCH_3)$_2$]	[Al(OH)(O_2CCH_3)$_2$] · 2 H_2O	[Al(OH)(O_2CCH_3)$_2$] · 1 CH_3OH	[Al(OH)(O_2CCH_3)$_2$] · 1 C_2H_5OH	[Al(OH)(O_2CCH_3)$_2$] · 1 $C_6H_5CH_3$
**Wavelength** (Å)	1.5406 Å				
**Crystal system**	monoclinic				
**Space group**	*Cc*	*C*2/*c*			
**Z**	/	2			/
**Unit cell dimensions** (Å), (°)	*a* = 13.887(3) Å, *b* = 12.007(3) Å, *c* = 12.314(3) Å, *α* = 90 °, *β* = 113.47(2) °, *γ* = 90 °	*a* = 13.9673(3) Å, *b* = 11.91431(19) Å, *c* = 12.0413(3) Å, *α* = 90 °, *β* = 119.3995(15) °, *γ* = 90 °	*a* = 14.1300(4) Å, *b* = 11.8249(2) Å, *c* = 12.1398(4) Å, *α* = 90 °, *β* = 117.448(2) °, *γ* = 90 °	*a* = 14.185(12) Å, *b* = 11.8537(7) Å, *c* = 12.1428(12) Å, *α* = 90 °, *β* = 118.939(6) °, *γ* = 90 °	*a* = 13.918(4) Å, *b* = 12.058(3) Å, *c* = 11.779(2) Å, *α* = 90 °, *β* = 118.90(15) °, *γ* = 90 °
**Volume** (Å^3)	1883.5(9)	1745.76(6)	1800.05(9)	1786.9(3)	1924.9(3)
R_{wp} (%)	3.95	6.40	3.60	9.34	7.59
**GoF** (%)	0.90	2.05	0.56	1.43	2.28

6. References

[1] N. Stock, *Microporous Mesoporous Mater.* **2010**, 9.
[2] M. O. Cichocka, J. Ångström, B. Wang, X. Zou, S. Smeets, *J. Appl. Crystallogr.* **2018**, *51*, 1652–1661.
[3] W. Kabsch, *Acta Crystallogr. D Biol. Crystallogr.* **2010**, *66*, 125–132.
[4] G. M. Sheldrick, *Acta Crystallogr. A* **2008**, *64*, 112–122.
[5] G. M. Sheldrick, *Acta Crystallogr. Sect. Found. Adv.* **2015**, *71*, 3–8.
[6] O. V. Dolomanov, L. J. Bourhis, R. J. Gildea, J. A. K. Howard, H. Puschmann, *J. Appl. Crystallogr.* **2009**, *42*, 339–341.
[7] B. Faddegon, J. Ramos-Méndez, J. Schuemann, A. McNamara, J. Shin, J. Perl, H. Paganetti, *Phys. Med.* **2020**, *72*, 114–121.
[8] J. Perl, J. Shin, J. Schumann, B. Faddegon, H. Paganetti, *Med. Phys.* **2012**, *39*, 6818–6837.
[9] P. H. Hsu, *Aluminum Hydroxides and Oxyhydroxides*, Soil Science Society Of America, USA, **1989**.
[10] Naegeli, *Verfahren Zur Herstellung von Normalem Neutralen Aluminiumacetat*, **1938**, 198966.
[11] K. C. Pande, R. C. Mehrotra, *Z Anorg Allg Chem* **1956**, *286*, 291–295.
[12] B. Achenbach, N. Stock, E. Svensson Grape, L.-M. Liedtke, A. K. Inge, *Kristalline Aluminiumdiacetat-Verbindungen, Kristalline Aluminiumdiacetat-Verbindung-Herstellungsverfahren Und Verwendung*, **2024**, DE 10 2024 111 075.9.
[13] B. Achenbach, E. S. Grape, M. Wahiduzzaman, S. K. Pappler, M. Meinhart, R. Siegel, G. Maurin, J. Senker, A. K. Inge, N. Stock, *Angew. Chem. Int. Ed.* **2023**, *62*, e202218679.
[14] G. Socrates, *Infrared and Raman Characteristic Group Frequencies: Tables and Charts*, Wiley, Chichester, **2010**.
[15] M. Ibrahim, E. Koglin, *Acta Chim Slov* **2004**.

Author Contributions

Synthesis screening, synthesis optimization, detailed characterization, including thermal analysis, IR spectroscopy as well as gas and vapor adsorption and powder X-ray diffraction including Rietveld-refinement for: B. Achenbach (compounds **1A, 1B, 3**) (data curation, formal analysis, investigation, validation), L. Liedtke (compound **2**) (data curation, formal analysis, investigation, validation).

Crystal structure determination by electron diffraction: E.S. Grape (data curation, formal analysis, investigation, validation), A. K. Inge (project administration)

Crystal structure determination by single crystal X-ray diffraction: C. Näther

Writing of original draft: B. Achenbach, L. Liedtke, N. Stock

Supervision and funding acquisition: N. Stock
